# Oxygenation, Life, and the Planetary System during Earth's Middle History: An Overview

**DOI:** 10.1089/ast.2020.2418

**Published:** 2021-08-16

**Authors:** Timothy W. Lyons, Charles W. Diamond, Noah J. Planavsky, Christopher T. Reinhard, Chao Li

**Affiliations:** ^1^ Department of Earth and Planetary Sciences, University of California, Riverside, California, USA.; ^2^ Department of Earth and Planetary Sciences, Yale University, New Haven, Connecticut, USA.; ^3^ School of Earth and Atmospheric Sciences, Georgia Institute of Technology, Atlanta, Georgia, USA.; ^4^ State Key Laboratory of Biogeology and Environmental Geology, China University of Geosciences, Wuhan, China.

**Keywords:** Biogeochemistry, Early Earth, Coevolving life and environments, Oxygen, Planetary systems, Complex life

## Abstract

The long history of life on Earth has unfolded as a cause-and-effect relationship with the evolving amount of oxygen (O_2_) in the oceans and atmosphere. Oxygen deficiency characterized our planet's first 2 billion years, yet evidence for biological O_2_ production and local enrichments in the surface ocean appear long before the first accumulations of O_2_ in the atmosphere roughly 2.4 to 2.3 billion years ago. Much has been written about this fundamental transition and the related balance between biological O_2_ production and sinks coupled to deep Earth processes that could buffer against the accumulation of biogenic O_2_. However, the relationship between complex life (eukaryotes, including animals) and later oxygenation is less clear. Some data suggest O_2_ was higher but still mostly low for another billion and a half years before increasing again around 800 million years ago, potentially setting a challenging course for complex life during its initial development and ecological expansion. The apparent rise in O_2_ around 800 million years ago is coincident with major developments in complex life. Multiple geochemical and paleontological records point to a major biogeochemical transition at that time, but whether rising and still dynamic biospheric oxygen triggered or merely followed from innovations in eukaryotic ecology, including the emergence of animals, is still debated. This paper focuses on the geochemical records of Earth's middle history, roughly 1.8 to 0.5 billion years ago, as a backdrop for exploring possible cause-and-effect relationships with biological evolution and the primary controls that may have set its pace, including solid Earth/tectonic processes, nutrient limitation, and their possible linkages. A richer mechanistic understanding of the interplay between coevolving life and Earth surface environments can provide a template for understanding and remotely searching for sustained habitability and even life on distant exoplanets.

## 1. Introduction

Earth's early oxygenation history has long been expressed in terms of two fundamental steps: the Great Oxidation Event (GOE) around 2.4 to 2.3 billion years ago (Ga) and a later rise in ocean-atmosphere oxygen levels between 800 and 600 million years ago (Ma) (Canfield, 2005; Kump, 2008; Lyons *et al.,* 2014). By some estimates, the onset of the GOE was marked by atmospheric O_2_ levels that were still relatively low and variable (*e.g.,* Poulton *et al.,* 2021). Whether this transition was a product of increasing O_2_ release to the biosphere or decreasing O_2_ sinks is debated (reviewed in Krissansen-Totton *et al.,* 2021). It was likely followed, however, by a dramatic increase in O_2_ inputs to the biosphere predicted from a large and long-lived anomaly in the carbon isotope record attributed to enhanced organic carbon burial (the Lomagundi-Jatuli Event; *e.g.,* Krissansen-Totton *et al.,* 2015). Interpretations of this feature point to potentially high levels of O_2_ in the atmosphere (*e.g.,* Bekker and Holland, 2012) and/or oxidized species (such as sulfate) in the oceans (Blättler *et al.,* 2018). Following the Lomagundi-Jatuli Event, atmospheric O_2_ levels fell (*e.g.,* Ossa Ossa *et al.,* 2018; compare Mänd *et al.,* 2020), ushering in the low-to-intermediate levels that would characterize the next billion years of Earth's history and creating very different environmental conditions for the beginnings of complex life.

Historically, the second rise has been linked to increasing diversity and complexity among eukaryotic organisms generally, expressed in part through increasing multicellularity and the appearance and diversification of animals specifically. In the absence of a cohesive geochemical argument for the second rise in oxygen, however, the record of the earliest animals is often treated as evidence for that oxygenation. In part because of this circular reasoning, the relationship between rising biotic complexity and the oxygenation of the ocean-atmosphere system has come under close scrutiny, producing challenges to the conventional view that increasing oxygen triggered the emergence of animals (reviewed in Cole *et al.,* 2020). A community of researchers is now reconsidering this time-honored link, some arguing that evolutionary patterns simply reflect the prolonged time scales necessary for genetic evolution of complex eukaryotes. The assertion that oxygen was not a factor controlling the timing of eukaryotic diversification builds from the view that oxygen was sufficiently high to support animals hundreds of millions of years before they appear in the rock record (Butterfield, 2009). Contributing to this viewpoint are suggestions that the earliest animals (Mills *et al.,* 2014) and eukaryotes more generally (Mentel and Martin, 2008) required only low levels of oxygen. Other challenges lie with the possibility that prevailingly low marine oxygen levels extended into the Paleozoic, well beyond the point where animals rose to ecological importance (Sperling *et al.,* 2015a).

Most recently, major improvements in our ability to track small-scale changes in oxygen levels in the ocean and atmosphere through time, reinforced by new biogeochemical modeling, suggest extreme variability with low baseline surface oxygen levels throughout much of Earth's history. The foundations of this new research include evidence for very low oxygen conditions in the atmosphere and oceans during the key middle chapters of Earth's history (∼1.8 to 0.8 Ga) followed by an abrupt rise of oxygen beginning around 800 Ma (Planavsky *et al.,* 2014). Despite the convergence of many data sets suggesting change at ∼800 Ma, an essential caveat is that correlations among environmental and evolutionary changes need not imply causation—and even if they do, challenges in distinguishing the causes from the effects can foster disagreement. Further, beyond relationships between environmental and biological coevolution, central questions remain about how our planetary system sustained clement surface conditions and modulated its atmosphere in ways that may or may not have been remotely detectable. Thus, our planet's history of accumulation of potential biosignature gases and changes to Earth's atmospheric greenhouse, viewed in the context of the collective drivers and feedbacks, informs our search for habitability and life on exoplanets (Schwieterman *et al.,* 2018).

## 2. Early Records of Complex Life

**FIG. 1.**
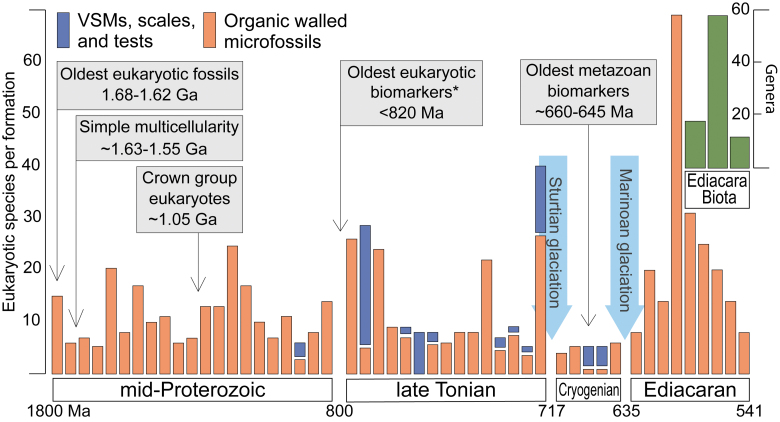
Eukaryotic microfossil diversity through time. Data include all formations of mid-Proterozoic through Ediacaran age for which greater than five eukaryotic species have been identified (updated from Cohen and Macdonald, 2015). Orange bars indicate diversity in organic-walled microfossils; blue bars indicate diversity of vase-shaped microfossils (VSMs), mineralized scales, and tests. Formations are arranged chronologically and separated into time periods of interest as discussed in the text (horizontal axis is not a linear scale). Diversity of Ediacaran animals defined in terms of global groupings based on three well-documented assemblages (Avalon, White Sea, and Nama; Erwin *et al.,* 2011). Supporting details are provided in the supplementary material (see Supplementary Fig. S1). *Oldest passing recent scrutiny; see Brocks *et al.* (2017) and text.

We focus on complex life here because all or most of the key prokaryotic metabolisms, including oxygenic photosynthesis, methanogenesis, and oxidative and reductive cycling of nitrogen and sulfur, developed much earlier (Lyons *et al.,* 2015). These pathways continued to influence and even dominate life in the oceans throughout Earth's middle history, and the primary controls on their impacts on the biosphere are repeated themes in this contribution. With that said, the most profound milestones specific to this interval lie with the rise and proliferation of algae, animals, and other complex life and with the cause-and-effect relationships between these evolutionary breakthroughs and environmental change. We can imagine, for example, time intervals with mostly hostile environments that stifled eukaryotic proliferation and minimized ecological impact, while at other times environmental variability and heterogeneities could have driven innovation. Landmarks in the development of complex life, for the purposes of this review, lie with several critical transitions in evolutionary history: the first physical (fossil) and geochemical (organic biomarker) records of eukaryotic life, crown group emergence and the earliest expressions of multicellularity among the eukaryotes, dramatic diversification and increasing abundances among the eukaryotes and associated ecological innovations, the first physical and geochemical signs of animal (metazoan) life, and innovation and ecological impact among animal lineages (Fig. 1).

We choose to emphasize the fossil and biomarker evidence for life transitions, rather than molecular (phylogenomic) approaches, because it is more straightforward to merge observable records of organismal presence, diversity, abundance, and ecology to coeval geochemical archives of environmental change, sometimes captured in the same samples. Such an approach sharpens our focus on the environmental significance of macroevolutionary lags—that is, the lag between the evolutionary origins of clades and later substantial, easily recognized contributions to global ecology (Erwin *et al.,* 2011). With that said, increasingly refined efforts to integrate “omic” perspectives of biotic evolution with independent fossil records (Peterson *et al.,* 2007) and geochemical archives of environmental change (Dupont *et al.,* 2006; Magnabosco *et al.,* 2018) have and will continue to yield essential fruit.

Clearly, delineating the timing of major evolutionary changes is sensitive to increasingly more sophisticated genomic approaches and dissections of the fossil record, yet the critical take-home message remains the same: key milestones in biological novelty came hundreds of millions of years prior to their ecological consequences. Some phylogenetic arguments permit extension of eukaryotic antiquity as deep into history as the beginnings of bacteria and archaea (Pace, 1997; compare Parfrey *et al.,* 2011); however, the first observable, datable, probable eukaryotic remains occur at ∼1.7 to 1.6 Ga (Knoll and Nowak, 2017; Javaux and Lepot, 2018)—consistent with molecular clock estimates that place the last common ancestor of extant eukaryotes between ∼1.87 and 1.68 Ga (Parfrey *et al.,* 2011; Betts *et al.,* 2018). Until recently the earliest organic biomarker evidence for eukaryotic sterol synthesis was dated to 2.7 Ga (Brocks *et al.,* 1999), but those records have now been convincingly tied to contamination (French *et al.,* 2015).

Eukaryotic multicellularity at ∼1.6 Ga followed soon after those 1.7 to 1.6 Ga stem eukaryote remains (Zhu *et al.,* 2016), and emergence of the crown group, expressed in simple multicellular red algae, is placed at ∼1.05 Ga (Butterfield, 2000; Gibson *et al.,* 2018) and more recently with reports of macroscopic multicellular green algae at ∼1.0 Ga (Tang *et al.,* 2020). Very recent evidence from phylogenetic analysis targeting sterol biosynthesis, an O_2_-requiring process, places the stem eukaryote split around the GOE (Gold *et al.,* 2017), which may not be a coincidence, and a recent fossil study argues for crown group (red algae) at 1.6 Ga (Bengtson *et al.,* 2017).

Perhaps of greatest relevance to defining the link between oxygen and biological evolution are the changes among eukaryotes observed in the fossil record at ∼800 to 750 Ma, including increasing diversity (Fig. 1) (reviewed in Knoll, 2014; Cohen and Macdonald, 2015; Riedman and Sadler, 2018). The mid-Proterozoic fossil record contains a total diversity of 30–40 very long-ranging (hundreds of millions of years) eukaryotic taxa, comprised exclusively of organic-walled microfossils, with individual assemblages rarely containing more than 10–15 species (reviewed in Cohen and Macdonald, 2015). While diversity among the organic-walled microfossils apparently decreased in the later Tonian (800–720 Ma), this deficit was more than made up for by the first appearances of vase-shaped microfossils (interpreted to have preyed on other eukaryotes; Porter, 2016) and scales (defensive structures), both at relatively high diversity.

If interpreted correctly, the vase-shaped microfossils, linked to testate amoebae, would be the earliest known example of eukaryophagy (protists preying on other protists). Recent work, using less aggressive separation techniques, hints at higher diversity within individual mid-Proterozoic formations (*e.g.,* Sergeev *et al.,* 2016; Beghin *et al.,* 2017); however, these new findings do not greatly expand the known diversity of pre-Tonian eukaryotes. Further, the development of eukaryotic predator-prey relationships remains one of the first significant evolutionary steps after nearly a billion years of relative stasis, with relevance to drivers of diversification (reviewed in Cohen and Macdonald, 2015) and as a possible consequence of environmental change. An equally intriguing possibility lies with claims for the earliest reliable organic biomarkers for ancient eukaryotes around 800 Ma (Brocks *et al.,* 2016, 2017; Zumberge *et al.,* 2020; and references cited therein), despite reports of earlier occurrences that would instead reflect contamination based on newer work. These new studies have taken exceptional steps, analogous to those described in the work of French *et al.* (2015) for Archean samples, to screen for secondary overprints. Such old samples, which have seen long and often complicated burial, collection, and storage histories, demand particular care. This need is exacerbated by the possibility of very low levels of primary steranes in samples deriving from a prokaryote-dominated world.

Cryogenian-aged (specifically 717 to 635 Ma) organic biomarkers from sponges found in samples from Oman (Love *et al.,* 2009; Love and Summons, 2015; Zumberge *et al.,* 2018) and possible sponge fossils of similar age (Maloof *et al.,* 2010) are the first traces of animal life, although a recent analysis of phylogenomic data suggests relatively rapid radiation of crown-group non-bilaterian animals in the interval leading up to the Cryogenian (Dohrmann and Wörheide, 2017). The Cryogenian is defined by the onset of snowball Earth glaciation at 717 Ma (Rooney *et al.,* 2015). Further, possible animal (sponge) organic biomarkers have been identified in the same pre-Cryogenian time slice (Brocks *et al.,* 2016, 2017; Zumberge *et al.,* 2020).

We then jump to the post-glacial Ediacaran (635 to 541 Ma), with macroscopic body fossils at ∼570 Ma (Pu *et al.,* 2016; rangeomorphs often, though not universally, assumed to be animals) followed by a great diversity of often large, dominantly soft-bodied animals, including bilaterians, and parallel advances in ecological complexity between ∼570 Ma and the Precambrian-Cambrian boundary (reviewed in Droser *et al.,* 2017). Those ecological innovations include motility, burrowing, and heterotrophy—although pervasive deep mixing of sediments would not be seen until well into the Paleozoic, more than 100 million years after the Ediacaran (Tarhan *et al.,* 2015). Despite the extraordinary array of early animals and lifestyles on display, the Ediacaran body fossils are limited to the interval between ∼570 Ma and the base of the Cambrian roughly 30 million years later (reviewed in Xiao and Laflamme, 2009; Droser *et al.,* 2017; Darroch *et al.,* 2018).

## 3. The Boring Billion

**FIG. 2.**
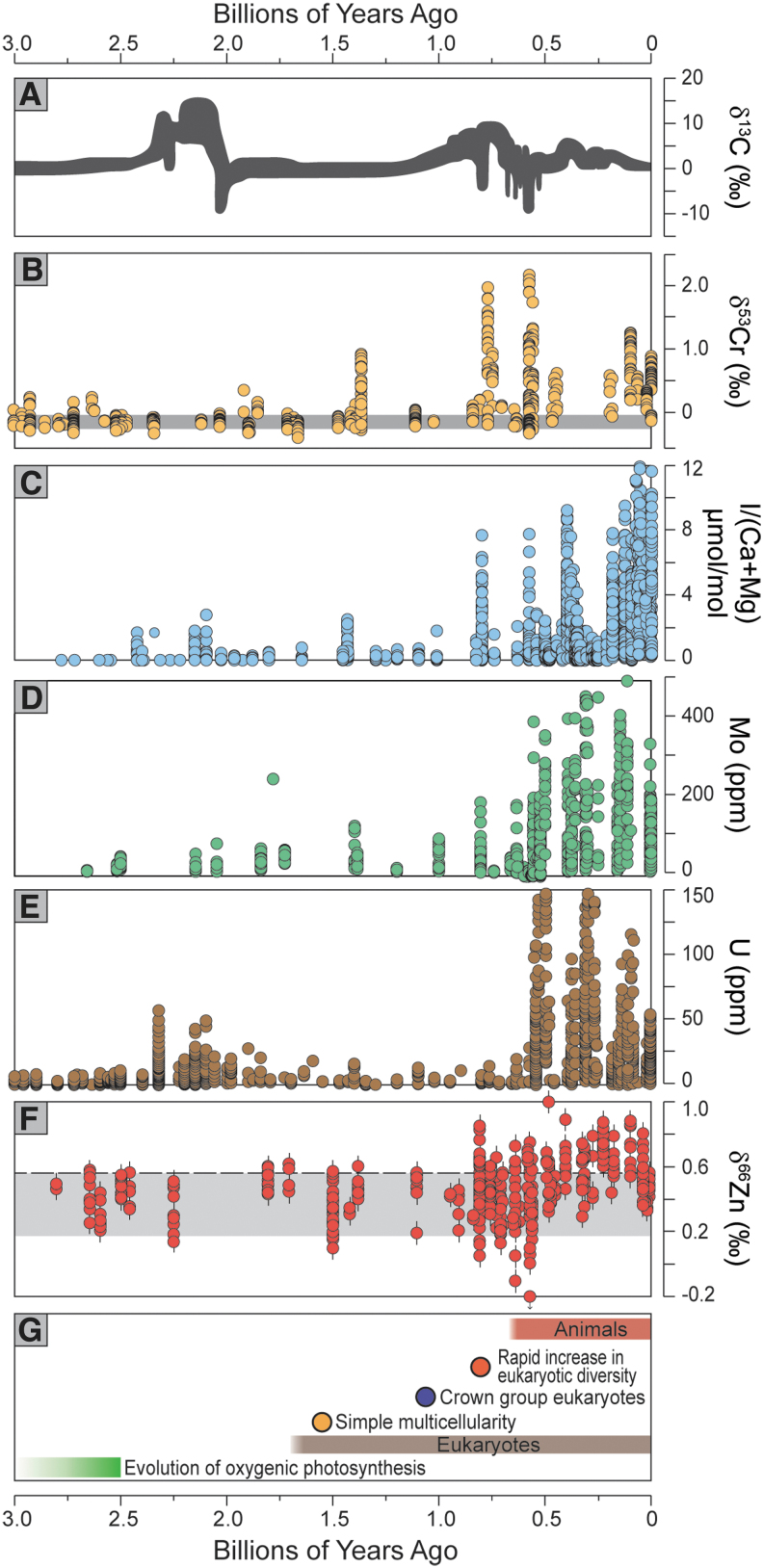
Summary of geochemical proxy records. (**A**) Carbon isotope data from marine carbonates (data from many sources); (**B**) chromium isotope data from marine ironstones and shales (Cole *et al.,* 2016; Canfield *et al.,* 2018); (**C**) iodine concentrations from shallow marine carbonates (Hardisty *et al.,* 2017; Lu *et al.,* 2018); (**D**) molybdenum (Scott *et al.,* 2008; Reinhard *et al.,* 2013) and (**E**) uranium concentrations (Partin *et al.,* 2013) from marine shales deposited under euxinic and anoxic conditions, respectively; (**F**) zinc isotope data from marine sedimentary sulfides (Isson *et al.,* 2018); and (**G**) timeline of evolutionary milestones. Carbon isotope data are interpreted at the first order to reflect the balance of organic-C to carbonate-C burial through time, with heavier values potentially reflecting higher organic-C burial and commensurate release of O_2_. Chromium isotope data are interpreted to be a direct measure of atmospheric O_2_ because large fractionations, like those beginning ∼800 Ma, require oxidative Cr-cycling in the presence of Mn-oxides during weathering and *p*O_2_ > 1% PAL (Planavsky *et al.,* 2014; Cole *et al.,* 2016). Iodine concentrations in carbonate rocks scale proportionally with concentration of iodate (the oxidized species of I) in seawater; higher concentrations beginning ∼800 Ma are interpreted to reflect increase in stability of well-oxygenated surface ocean conditions and concomitant deepening of the chemocline (Hardisty *et al.,* 2017). The concentrations of Mo and U in marine shales scale proportionally with the concentrations of these elements in overlying seawater. The reservoir sizes of these redox-sensitive elements are modulated at first order by the spatial extent of reducing bottom-water conditions (and hence the size of the global removal flux), with expansion of oceanic euxinia and anoxia drawing down concentrations of Mo and U, respectively. The increase in Mo at ∼800 Ma is therefore interpreted to represent a decrease in the prevalence of euxinia. A corresponding increase in U concentrations has not been observed, suggesting that the event recorded in other proxies at ∼800 Ma could have been restricted to the atmosphere, shallow oceans, and intermediate depths along continental margins where euxinia prevailed—while the deep ocean remained stably anoxic until later times. A delay in the rise of phosphorus and rhenium, like that observed for uranium, could also be explained by later deep-water oxygenation (Reinhard *et al.,* 2017a; Sheen *et al.,* 2018). Supporting details are provided in the supplementary material.

Understanding any relationships between oxygen and biological evolution requires that we explore the full time-interval over which eukaryotes first appeared, diversified, grew in numbers, and gave rise to the observed animal record. This time slice, broadly between 1.8 and 0.8 Ga, is known informally as the mid-Proterozoic. Despite its importance for the origins of eukaryotes, this middle chapter in Earth's history was once described as the “dullest time in Earth's history” owing to the observed uniformity of carbon isotope compositions for carbonate rocks of this age (Buick *et al.,* 1995), particularly when contrasted with the striking isotopic variability that characterizes the intervals immediately before and after—arguably the most dramatic in Earth's history (Fig. 2). Because these isotope data are widely thought to be linked mechanistically to rates of organic carbon burial, the major driver of biospheric oxygenation, this billion-year period was imagined to have been equally “dull” in terms of atmospheric and oceanic oxygenation. With further work, the spatial and temporal persistence of this carbon isotope pattern has been reinforced, suggesting a “prolonged period of stability in crustal dynamics, redox state of surface environments, and planetary climate” (Brasier and Lindsay, 1998) to the degree that it is now widely referred to as the “boring billion.” We will continue to use this term because of deeply entrenched historical precedent.

In our discussion of oxygen's relationship with evolving life, habitats in the shallow parts of the ocean are most important, but the character and consistency of conditions in those waters is intimately related to the atmosphere above and the deep ocean below. Early estimates for stable atmospheric oxygen levels during the boring billion converged on a range from 1% to 40% of the present atmospheric level (Kump, 2008) (PAL; O_2_ presently comprises 21% of the atmosphere) until recent suggestions of even lower possible concentrations (addressed below).

The lower limit of 1% was based on oxidative weathering and specifically the level of O_2_ required to retain insoluble iron [Fe(III)] in ancient soils, which has been described from this time—in contrast to the loss of Fe in soils before the GOE (Rye and Holland, 1998). In truth, however, this approach is strongly reliant on independent estimates of atmospheric CO_2_ concentrations and assumptions about Fe oxidation kinetics and soil hydrology, which are equally fraught with uncertainty, and thus substantially lower or higher estimates for atmospheric O_2_ are possible from this method. More importantly, however, the best preserved mid-Proterozoic soils actually do bear signs of Fe loss during weathering, suggesting lower O_2_ (Mitchell and Sheldon, 2010).

The upper estimate of 40% PAL was grounded in something entirely different—maximum levels of O_2_ in the atmosphere that would have allowed the deep ocean to remain anoxic (Canfield, 1998), which is now well accepted for the boring billion (see discussions below). The estimate of 40% ultimately lies with a simple box model for O_2_ accumulation in the deep ocean and its relationship to O_2_ levels in the atmosphere and carries assumptions about patterns and efficiency of O_2_ mixing into and through the oceans, nutrient inventories, and O_2_ consumption via organic decay in the deep waters below the photic zone. Subsequently, the upper limit for the estimate was dialed down to something closer to 10% (Kump, 2008), and a recent rethink of the phosphorus assumptions employed in the landmark Canfield contribution (Derry, 2015) reduced the estimate for atmospheric *p*O_2_ to far less than 10% PAL. These and related details are discussed further below.

**FIG. 3.**
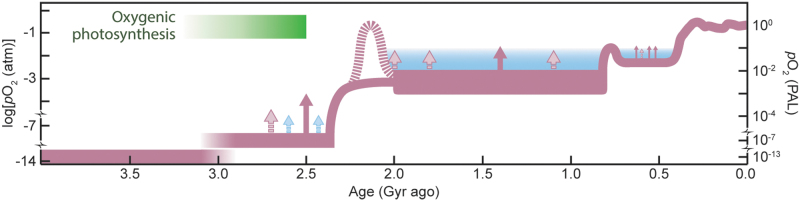
Evolution of Earth's atmospheric oxygen content through time. Red curve shows the authors' preferred model for long-term evolution (see text for details), while the blue shaded region reflects alternative views based on numerical simulations that predict O_2_ stability only at higher *p*O_2_ (Daines *et al.,* 2017). Solid red arrows denote possible transient increases in *p*O_2_ for which geochemical evidence exists; pink dashed arrows indicate less certain events. Blue arrows in the late Archean indicate hypothetical “whiffs” of O_2_ and are included to highlight schematically the oscillatory system behavior assumed to accompany transitions from one steady state to another. Supporting details are provided in the supplementary material (see Supplementary Fig. S2). PAL = present atmospheric level.

Nonetheless, Canfield's (1998) arguments for protracted anoxia in the deep ocean long after O_2_ began to accumulate in the atmosphere helped define a generation of research, demanding a search for geochemical evidence and possible biological consequences. First and foremost, protracted anoxia fundamentally challenged the previous assumption that the full ocean was O_2_-rich during the boring billion (Wilde, 1987; Canfield, 1998) based on the disappearance of anoxia-dependent iron formations at ∼1.8 Ga (Fe is soluble only in O_2_-free waters). Reconciling the disappearance of iron formations with prolonged anoxia led instead to the idea that accumulation of hydrogen sulfide (H_2_S) was responsible for the loss of iron formations at the start of the boring billion (Canfield, 1998). Iron is insoluble in anoxic waters when they are rich in H_2_S (an ocean state known as *euxinia,* which dominates the Black Sea but is otherwise rare in the ocean today). The pattern of stable, likely intermediate levels of atmospheric O_2_ during the boring billion stands in contrast to the dramatic rising and falling levels that characterized the preceding GOE and the Neoproterozoic Oxidation Event (NOE; Och and Shields-Zhou, 2012) that followed (Fig. 3; reviewed in Lyons *et al.,* 2014). The Proterozoic spanned from 2.5 to 0.54 Ga, with the Neoproterozoic beginning at 1.0 Ga.

Details from the boring billion suggest climatic stability expressed in an unusual, if not unique, lack of widespread glaciation despite the muted warming of a still faint sun and the records of the world's greatest (snowball Earth) glaciations in the intervals before and after. Plate tectonics was in full swing (Cawood, 2020), but as another testament to the unique stability of this time, the tectonic/orogenic signals are far less dramatic than those before and after (Tang *et al.,* 2021)—marked instead by lithospheric stability and a paucity of both passive margins and geochemical records of mountain building and intense concomitant weathering (Cawood and Hawkesworth, 2014). In apparent contradiction to these observations, however, a supercontinent did form, as reviewed by Roberts (2013; see also Evans, 2013, and Cawood and Hawkesworth, 2014). The paucity of geochemical signatures may thus reflect muted intensity rather than the absence of tectonic activity. The supercontinent Nuna (also known as Columbia) was assembled by the beginning of the boring billion and remained relatively intact for hundreds of millions of years. Nascent rifting followed, particularly at ∼1.4 Ga, but the continental mass mostly retained its unified integrity rather than experiencing widespread dispersal of continental fragments. With little modification, this was followed by reassembly into Rodinia roughly a billion years ago. Again, stability and specifically subdued tectonic/orogenic drivers (Tang *et al.,* 2021) may have been a defining quality, although details of Nuna breakup are not well known, and more dramatic dispersal is possible (as reviewed in Planavsky *et al.,* 2015; Pehrsson *et al.,* 2016; Salminen *et al.,* 2020).

One could argue that the truly remarkable observation about the boring billion is that it is bracketed by some of the most striking transitions in Earth's history—expressed in oxygen, life, climate, and first-order tectonic reorganization. As such, Earth's middle chapter is both a consequence and harbinger of change, yet it reveals itself through its stability as being the opposite. It is clear to us that such a pattern of steadiness and the bookends of change on either side are not easily explained—but are anything but boring.

## 4. New Perspectives

It is possible that the boring billion interval offered a challenging O_2_ landscape for early complex life until its end, around 800 Ma. This transition is marked by strong temporal correlation among eukaryotic innovation, records of the earliest animals, and environmental change expressed in multiple proxies. It follows that ocean oxygenation may have been a critical step required for the radiation of complex, eukaryote-dominated ecosystems—O_2_ is certainly a prerequisite for their existence on the modern Earth. Confidence in this assertion, however, depends largely on the latest opinions about O_2_ during the boring billion and its immediate aftermath.

The aforementioned claim of persistent deep-ocean anoxia during the boring billion (Canfield, 1998) has gained a strong footing through detailed analysis of fine-grained sedimentary rocks and the use of well-established geochemical tracers. Specifically, the Fe chemistry of shales can delineate local anoxic conditions and even the presence of hydrogen sulfide in the water column as witnessed by the extent of associated conversion to pyrite (FeS_2_). Distributions of trace metals sensitive to the redox conditions of their surroundings, such as molybdenum and uranium, and their isotopic signatures can bring complementary value by giving us a global view. Temporal patterns among these trace metals in marine shales are controlled by the sizes of their reservoirs in global seawater, which are in turn modulated by the overall redox landscape of the oceans and lower solubilities of the metals under anoxic and euxinic conditions. Based on the collective data for the boring billion (Arnold *et al.,* 2004; Scott *et al.,* 2008; Planavsky *et al.,* 2011; Poulton and Canfield, 2011; Partin *et al.,* 2013; Reinhard *et al.,* 2013; as reviewed in Lyons *et al.,* 2014), Fe-rich (ferruginous) waters must have dominated the deeper ocean. Areas of the seafloor bathed in bottom waters containing hydrogen sulfide were likely restricted to productive portions of ocean margins. Although limited in extent, euxinia then was likely two orders of magnitude more extensive than in today's oceans where it blankets much less than 1% of the seafloor (Reinhard *et al.,* 2013).

Some recent approximations of atmospheric O_2_ during the boring billion fall far below conventional estimates (Fig. 3). In particular, chromium (Cr) isotope data for the boring billion suggest *p*O_2_ levels of <1% PAL and perhaps as low as 0.1% PAL or less (Crowe *et al.,* 2013; Planavsky *et al.,* 2014), thus challenging the traditional view of 1% to 10% PAL (and as high as 40%). Details of the Cr isotope method are provided elsewhere (Frei *et al.,* 2009; Planavsky *et al.,* 2014), but there are three essential take-home messages as follows: (1) Cr isotope fractionation is typically attributed to coupled manganese (Mn) oxidation in soil profiles, a process that requires a critical threshold of surface oxygen; (2) Fe must be quantitatively oxidized within the soil profile such that isotopically fractionated Cr can escape the soil; and (3) these are difficult estimates to make because of the challenges intrinsic to using soil redox proxies, or the siliciclastic archives that were sourced by those soils and accumulated downstream, as quantitative windows to the overlying atmosphere (Daines *et al.,* 2017). Nevertheless, the signatures of these soil processes can potentially be captured in marine sediments, and corroborating evidence for these relationships was recently reported from ancient soil horizons spanning the same time interval (Colwyn *et al.,* 2019). Further research will determine the veracity of the recent *p*O_2_ estimates, bolstered by continued use of well-established models for Fe oxidation in ancient soils. Regardless of any remaining uncertainties surrounding the *p*O_2_ estimates, many of the Cr isotope data are strikingly unfractionated during the boring billion, suggesting fundamentally different biospheric redox relative to the interval that followed (Fig. 2). These hypothesized low O_2_ levels are consistent with a fresh view of the paleosol data (Planavsky *et al.,* 2018), including mid-Proterozoic soils that lost Fe during weathering—the oft-cited paleobarometer of atmospheric *p*O_2_.

Likely of greatest importance in our discussion were the redox conditions in the shallowest oceans during the boring billion when the earliest eukaryotes were finding their first footholds. Ironically, these biologically critical settings have been assessed only rarely because standard geochemical methods are aimed instead at deeper marine waters and the atmosphere. One remedy may lie with the iodine content of carbonate rocks. Iodide (I^-^) in seawater oxidizes to iodate (IO_3_^-^) under low (micromolar) levels of dissolved oxygen similar to those that define the low end of eukaryotic tolerance. Importantly, iodate but not iodide readily substitutes into carbonate rocks and can fingerprint the presence of O_2_ in ancient marine waters (Hardisty *et al.,* 2017). The record for the boring billion reveals a persistence of low iodate levels in shallow water settings, suggesting the presence of oxygen (Fig. 2; Hardisty *et al.,* 2017). However, the low values are most reminiscent of those seen today on the margins of oxygen minimum zones—reflecting the mixing between proximal oxic and anoxic waters and the slow reaction kinetics of iodide-to-iodate oxidation. Cerium (Ce) records from the mid-Proterozoic point similarly to low oxygen conditions even in shallow waters where carbonates were forming (Tang *et al.,* 2016; Liu *et al.,* 2021).

We can reasonably interpret the record of the boring billion as reflecting a shallow chemocline at the upper boundary of deep anoxic waters with frequent upward mixing and consequent low and unstable O_2_ in the surface oceans. If correct, episodic upward intrusion of toxic H_2_S may have swamped ocean margins. Such conditions would have permitted, yet at the same time frequently challenged, O_2_-dependent life in the shallow ocean.

Recent three-dimensional modeling of redox landscapes in the ancient oceans provides additional perspective on the patterns and mechanisms of oxygenation and habitability during the boring billion (Reinhard *et al.,* 2016). Even at levels as high as a few percent of present-day atmospheric O_2_, we can expect disequilibrium between the atmosphere and the local, photosynthetically sourced O_2_ of the shallow ocean, resulting in spatial concentration heterogeneities (O_2_ oases in the upper ocean) in regions of intense upwelling and thus highest biological production. The models further predict a general absence of O_2_ in bottom waters except perhaps in the shallowest regions. This constraint matters most to the earliest animals, which are known to have been benthic. Simpler calculations, as informed in a general sense by processes occurring in the modern shallow oceans, also predict pronounced temporal (seasonal) variability in O_2_ production and accumulation. These model results from the work of Reinhard *et al.* (2016), together with the iodine and cerium data, thus suggest an O_2_ landscape that may have been challenging for early eukaryotic life—and one that may have delayed the first animals (see Cole *et al.,* 2020, for contrasting views).

The possibility of a world with atmospheric *p*O_2_ <0.1–1% PAL demands thorough consideration of the patterns and controls on primary production, nutrient availability, carbon cycling, and related stabilizing forcings and feedbacks. These deliberations distil down to the critical O_2_ sources and sinks, and in particular whether and how they respond to ambient ocean-atmosphere O_2_ levels in ways that provide negative feedback or promote instability. For example, it is widely accepted that a decrease in atmospheric O_2_ levels should, in general, lead to an increase in organic matter burial (and thus O_2_ release), while also leading to a decrease in organic matter weathering and thus reduced O_2_ consumption (Laakso and Schrag, 2014). As a result, there is widespread agreement that the most effective mechanism for stabilizing low atmospheric O_2_ is reducing global rates of biospheric productivity (Laakso and Schrag, 2014; Derry, 2015; Reinhard *et al.,* 2017a). Indeed, the stability of the carbon isotope curve—hovering around zero per mil (Fig. 2)—contributed to the initial definition of the boring billion (Buick *et al.,* 1995; Brasier and Lindsay, 1998) and attests to a relatively low steady-state background of organic burial and thus O_2_ release during this interval.

Nearly two decades ago the idea of limited organic production and burial during the boring billion gained momentum with the publication of Anbar and Knoll (2002). The authors developed the concept of nutrient throttling, specifically through low availability of trace elements critical to major microbial enzymatic pathways—most notably molybdenum's role as a cofactor in nitrogenase during nitrogen (N) fixation (Anbar and Knoll, 2002). Muted metal enrichments in euxinic shales support the possibility of low marine molybdenum (Mo) inventories during the boring billion due to high rates of sediment uptake within the anoxic and euxinic oceans (Scott *et al.,* 2008), but sensitivity of the N cycle and specifically N_2_-fixing microbes to Mo availability remains a hot topic of research (Glass *et al.,* 2012; Zerkle and Mikhail, 2017). Extensive microbial denitrification in the broadly anoxic waters of the ocean would have contributed to fixed-N limitation during the boring billion (Reinhard *et al.,* 2017a), although this effect might have been muted under the Fe-rich conditions of those waters (Michiels *et al.,* 2017). It remains unknown whether, and to what degree, scarcity of Mo and other trace metals limited N bioavailability and primary production more generally during the boring billion (Reinhard *et al.,* 2013; Large *et al.,* 2018).

A recurring theme in these discussions is the relationship between the likely persistence of anoxia in the deep oceans during the boring billion and impacts on nutrient cycling. Recent efforts have shifted much of the conversation to phosphorus(P)-based controls on primary production and associated oxygenation during this time window (Ozaki *et al.,* 2019; Canfield *et al.,* 2020). Central to these arguments is the coupling between P and Fe cycling in widely ferruginous oceans—as specifically related to Fe-P mineral formation and the strong capacity for P scavenging by Fe oxides (Laakso and Schrag, 2014; Derry, 2015; Reinhard *et al.,* 2017a; Guilbaud *et al.,* 2020; compare Johnson *et al.,* 2020). Other models note important contributions to P limitation from muted recycling of P under anoxic marine conditions (Kipp and Stüeken, 2017; Laakso and Schrag, 2018, 2019). The extent of P limitation remains a critical question. Intense throttling of biospheric productivity by P deficiencies can strongly reduce biospheric N demand and thus commensurately decrease the importance of N fixation. Regardless, it appears that primary production during the boring billion was limited—as supported by recent novel triple oxygen isotope measurements (Crockford *et al.,* 2018; Hodgskiss *et al.,* 2019). These low levels of production were likely stabilized by P limitation but with potential reinforcement by transient N limitation during attempted excursions to higher *p*O_2_ (Reinhard *et al.,* 2017a). Implicit to a world dominated by P limitation is the importance of tectonic and volcanic activity that can enhance P delivery to the oceans via weathering. In sum, a number of avenues seem to be converging on a generally low-P, high-Fe, and low-O_2_ Earth system state for much of Proterozoic time with limited biospheric productivity (Planavsky *et al.,* 2018).

Other recent research highlights the importance of concomitant O_2_ sinks linked to oxidation of reduced species on land and in the oceans—compounds bearing reduced C, sulfur (S), and Fe in particular, including organic matter, pyrite (FeS_2_), and dissolved S and Fe in seawater (Laakso and Schrag, 2014; Daines *et al.,* 2017). Relevant in this discussion is the degree to which the proxies and models are (Planavsky *et al.,* 2014) or are not (Daines *et al.,* 2017) consistent with stabilization of very low (∼0.1% PAL) O_2_ in the atmosphere. Specifically, recent model-based arguments assert that loss of stabilizing feedbacks at low atmospheric O_2_ challenges the possibility of a stable mid-Proterozoic atmosphere with O_2_ levels lower than 1% PAL. Levels higher than 1% PAL have also been suggested based on trace element contents of pyrite (Large *et al.,* 2019; Steadman *et al.,* 2020; Yierpan *et al.,* 2020). Uncertainties in model-based estimates lie with insufficiently known parameters such as sulfide oxidation rates and strong sensitivity to other poorly known environmental factors. At the same time, geochemical proxies, including the pyrite data, rely on similarly uncertain calibrations dependent on under-constrained model parameters. Further still, very low atmospheric O_2_ levels can be difficult to reconcile with expected photolysis of O_2_ in the lower atmosphere in the absence of sufficient ozone shielding (Goldblatt *et al.,* 2006). The good news is that all approaches point consistently to intermediate atmospheric *p*O_2_ during the boring billion, but the atmospheric *p*O_2_ estimates via competing methods can differ by as much as 2 orders of magnitude. No doubt these differences contribute to contrasting views of O_2_'s role in modulating the pace and pattern of evolution during the boring billion.

Another consequence of low and thus more easily perturbed levels of biospheric O_2_ is the possibility of oscillation around a quasi-stable baseline (Fig. 3), with dynamic *p*O_2_ favored during the boring billion by still relatively low *p*O_2_ in the atmosphere controlled by a complex, feedback-rich interplay between nutrients, tectonics, erosion, and the related weathering sinks. Despite the suggestions of overall low atmospheric and marine O_2_ during the boring billion, a growing body of research points to at least episodes of higher O_2_ (*e.g.,* Sperling *et al.,* 2014; Gilleaudeau *et al.,* 2016; Mukherjee and Large, 2016; Tang *et al.,* 2016; Hardisty *et al.,* 2017; Yang *et al.,* 2017; Planavsky *et al.,* 2018; Sheen *et al.,* 2018; Zhang *et al.,* 2018; Mänd *et al.,* 2020; Kendall, 2021; see also Zhang *et al.,* 2016, but compare Diamond *et al.,* 2018). It remains to be seen whether each is truly a manifestation of higher O_2_ in the atmosphere and global ocean and, if true, whether drivers such as solid-Earth processes and emplacement of large igneous provinces (LIPs) specifically (Diamond *et al.,* 2021) may have played a role. If oxygenation events were present and even prevalent during the boring billion, the question then becomes whether there were biological consequences, such as early multicellularity and development of many of the basic characteristics of eukaryotic life, as observed for example in the Roper Group of Australia at 1.4 Ga (Javaux and Knoll, 2017). The data are presently too sparse to illuminate the full scope of such records, and biases reflecting sample availability are a real concern. Nevertheless, a particularly rich archive of potentially more oxygenated conditions has emerged at roughly 1.4 Ga, including records from the Roper Group (Yang *et al.,* 2017)—right in the middle of the boring billion.

Dynamic redox at that time is certainly consistent with the notion that O_2_ levels were generally much lower than today's and were set and pulsed by the balance between production and consumption. Temporal variability may well help us reconcile seemingly conflicting reports of low (even very low) and higher *p*O_2_ over the same billion years of Earth history. Tectonic models during this interval as specifically related to rifting during breakup of the Nuna supercontinent at ∼1.4 Ga could provide a mechanism for periods of enhanced organic burial and related transient oxygenation (Zhang *et al.,* 2012; Canfield *et al.,* 2018; Diamond and Lyons, 2018; Large *et al.,* 2019; Steadman *et al.,* 2020). This and other possible oxygenation events could be more specifically related to activity in LIPs and concomitant enhanced delivery of nutrients to the oceans (P and trace metals in particular; Diamond *et al.,* 2021). Others have speculated on additional oxygenation events during the boring billion and possible relationships to tectonics/LIPs activity and life (*e.g.,* Campbell and Allen, 2008; Diamond and Lyons, 2018; Zhang *et al.,* 2018; Diamond *et al.,* 2021). Finally, the likelihood of redox heterogeneities in the oceans (Slack *et al.,* 2007; Sperling *et al.,* 2014; Reinhard *et al.,* 2016) tells us that coeval regions of more and less oxygenated waters may also have been a common feature during the boring billion.

## 5. A World of Change 800 Million Years Ago?

The conditions, causes, and consequences of the boring billion must rank among the most important and elusive in studies of early Earth and its life, but the more intriguing stories may lie at its end. It has long been assumed that the second major step in Earth's oxygenation history (the Neoproterozoic Oxidation Event, NOE) occurred late in the Proterozoic, in the window from roughly 800 to 600 Ma (Fig. 3). The vagueness of this age constraint, however, has left open the possibility that this NOE was either the cause or a consequence of the snowball Earth-scale glaciations that started around 717 Ma or, through various feedbacks, that both are true. It is not our goal to resolve or even fully articulate that debate, but we will outline evidence for important changes in both the environment and life at roughly 800 Ma and argue that these environmental changes could reflect extrinsic drivers—particularly the role of plate tectonics.

The list of geochemical and paleontological markers of change at roughly 800 Ma is long and growing—providing an easy target for defining the end of the boring billion period (Fig. 2). Foremost, the long run of stable carbon isotope (δ^13^C) values shows a marked shift to positive values (Fig. 2), suggesting a fundamental transition in C cycling likely related to increased organic carbon burial and associated O_2_ release to the biosphere (Krissansen-Totton *et al.,* 2015). The precise timing of this jump is not well known because of poor age control (Halverson and Shields-Zhou, 2011), but recent data place it in the interval immediately before the Bitter Springs δ^13^C anomaly (Macdonald *et al.,* 2010; Kuznetsov *et al.,* 2017). The Bitter Springs event, the onset of which has been well dated at ∼810 Ma (Macdonald *et al.,* 2010), is the first in a series of late Proterozoic negative isotope excursions, but it is preceded by obvious positive δ^13^C values that seem to define the end of the boring billion. Clearly, the “boring” isotope record of the preceding billion years had given way to dramatic variability, both positive and negative, intimately tied to C cycling and likely coupled to release and consumption of O_2_. Yet the mechanisms and implications of those carbon-oxygen relationships are beyond simple burial and oxidation of marine organic matter and remain topics of discussion.

Many other indicators suggest a fundamental shift in redox structuring around 800 Ma, including fingerprints of higher trace metal concentrations in seawater and elevated dissolved sulfate levels expressed by the first appearance of massive sulfate evaporite deposits worldwide (Thomson *et al.,* 2015; Turner and Bekker, 2016), which both likely record increased O_2_ in the oceans. Widespread anoxic conditions in the oceans such as during the boring billion, in contrast, favor burial of redox-sensitive metals and pyrite derived from microbial sulfate reduction. Similarly, Cr isotope data from shales show their earliest indications of increased atmospheric oxygenation (Planavsky *et al.,* 2014; Cole *et al.,* 2016). Selenium (Se) isotope data insinuate increasingly oxidizing conditions in the ocean starting around 770 Ma (Von Strandmann *et al.,* 2015), and iodine results in carbonate rocks suggest, for the first time, appreciable and sustained O_2_ at least locally in the surface ocean coincident with the Bitter Springs C isotope anomaly (Lu *et al.,* 2017). Consistent with these observations, more comprehensive Fe oxidation in the oceans in the *ca.* 800 Ma window is suggested in the Fe isotope record of ironstones (Wang *et al.,* unpublished data).

Oxygen likely took a step up in the ocean-atmosphere system, but it is hard to know how big that step was. Such constraints will be a popular area of future research, and new models will continue to emerge (*e.g.,* Kanzaki and Kump, 2017). Moreover, recent data for gas inclusions in halite dated at 815 Ma, interpreted to reflect trapped ancient atmosphere, put the rise to 50% PAL (Blamey *et al.,* 2016; also Steadman *et al.,* 2020). That number was brought down to less than 10% PAL in a reassessment of those data (Yeung, 2017), which might still have been a large increase depending on levels present during the boring billion. We should continue to refine the precision of the age relationships and the strength of our paleoredox proxies while developing coherent mechanistic models. Many of the observations are closer to suggestions than statistically robust trends (Cole *et al.,* 2020), and a lack of sufficient age controls challenges our ability to feel comfortable about the specific timing relationships among the proxies, their synchroneity, or possible cause-and-effect relationships. Nevertheless, wide-ranging signs seem to be pointing in the same direction.

There is a remarkable overarching temporal coincidence among the archives of biotic innovation and proliferation and the aforementioned geochemical expressions of environmental change, such as the striking transformation in C isotope behavior. Eukaryotic fossils show considerable taxonomic and ecological diversification at ∼800 to 750 Ma (reviewed in Knoll, 2014; Cohen and Macdonald, 2015; Riedman and Sadler, 2018), almost a billion years after their first known appearance. And, in what may be the most important tie point in this discussion, organic biomarker evidence for eukaryotic life (steranes), when vetted for miscues linked to contamination, does not appear until ∼820 Ma (Brocks *et al.,* 2017; Brocks, 2018; Zumberge *et al.,* 2020)—despite many searches in diverse paleoenvironmental settings for earlier occurrences (*e.g.,* Nguyen *et al.,* 2019). Consistent with these organic trends, zinc (Zn) isotope data, an emerging geochemical tracer, offer tantalizing hints of increasing eukaryotic biomass at ∼800 Ma (Isson *et al.,* 2018) supported by greater eukaryotic microfossil abundances in the same time window (Cohen and Macdonald, 2015). The long gap between the first appearance of fossils at about 1.7 to 1.6 Ga and reliable biomarkers almost a billion years later can reasonably be viewed as an expression of generally low eukaryote abundances (ecological significance) through most of the boring billion (Gold, 2018), assuming early synthesis of sterols (the precursors of the fossil steranes) by eukaryotes (Gold *et al.,* 2017, compare Porter, 2020). For example, early eukaryotes living under low O_2_ conditions may not have produced sterols (Takishita *et al.,* 2017). This is also consistent with models of relatively low nutrient P in the ocean interior, which may have limited the overall scope of Earth's eukaryotic biosphere despite the presence of eukaryotic life in the surface ocean (Reinhard *et al.,* 2020).

It has long been tempting to call upon the largest-scale tectonic processes to explain the NOE. Such propositions can be straightforward (compare Alcott *et al.,* 2019), and the breakup of Rodinia falls in just the right temporal window. Also, Rodinia breakup was far more decisive than the possibly half-hearted efforts of Nuna, and the relative consequences for ocean chemistry, nutrient cycling, and inevitably oxygenation may have differed commensurately. Li *et al.* (2008) noted widespread continental rifting between ∼825 and 740 Ma and episodic plume events at ∼825, ∼780, and ∼750 Ma. Rifted basins are efficient sediment traps and favor large-scale organic burial along continental margins. The plume events and associated LIPs (concentrated between 850 and 720 Ma) may have pumped out copious amounts of P via basalt weathering—stimulating primary production, organic burial, and gains in biospheric O_2_ (Horton, 2015, but compare Planavsky, 2018).

The possibility of increasing oxygenation prior to snowball Earth glaciation has invited some researchers to blame the dramatic cooling and onset of global-scale ice cover on the rise in O_2_—specifically through decreasing methane stability in the atmosphere (Pavlov *et al.,* 2003). Alternatively, methane may have mostly been much lower than previously imagined during the boring billion due to muted production and preservation in the oceans and photochemical instability in the atmosphere (Olson *et al.,* 2016). The balance of greenhouse gases that maintained a mostly warm, ice-free boring billion in the face of a still faint sun is a work in progress (Fiorella and Sheldon, 2017; Zhao *et al.,* 2018).

Still other studies highlight the importance of snowball Earth glaciation, through the sheer magnitude of related weathering processes (Hoffman *et al.,* 1998, 2017), as a trigger for high nutrient fluxes to the oceans, associated primary production, O_2_ release (Planavsky *et al.,* 2010; Sahoo *et al.,* 2012), and eukaryotic diversification (Brocks *et al.,* 2017). This is a classic chicken-or-the-egg argument, and many other controls are possible, but it is also reasonable to imagine both scenarios at play—that is, a one-two punch, with glaciation and its impact on O_2_ as a positive feedback set in motion by previous tectonically driven oxygenation. In other words, imagine that rising oxygen both triggered and benefited from large-scale glaciation, that O_2_ may have risen and fallen more than once during this interval, and that complex life would have been affected at each step along the way.

## 6. Evolutionary Lags, Oxygen Triggers, and the Emergence of Complex Life—A Synthesis

Eukaryotic life took big steps forward during the boring billion, but those advances have not been correlated with clear signatures of environmental change. Further, if animals were around during the boring billion, they left no fossils, biomarkers, or traces of their ecological impacts, and recent molecular clock studies agree on a Tonian origin (Erwin *et al.,* 2011; Dos Reis *et al.,* 2015; Dohrmann and Wörheide, 2017). Overall, early eukaryotes seemed content to continue their development in the absence of great diversity, abundance, and ecological consequence. Life, then, was in many senses experiencing its own kind of stasis through the boring billion, and the evolutionary novelties that did occur may or may not have derived from environmental drivers.

Some milestones, including the first expressions of eukaryotic life, multicellularity, and even the earliest animals, may instead owe their timing to the evolutionary pacing intrinsic to developmental-genetic toolkits rather than something extrinsic (Butterfield, 2009), as in oxygen or other major environmental factors. Alternatively, the external drivers may have been more subtly expressed (perhaps acting transiently) or less clearly related to oxygen specifically. Again, we are making the critical distinction between the beginnings of eukaryotic life as recorded in the geologic record and their later proliferation and innovation to the point of profoundly impacting ecosystems.

The earliest animals have captured much of the attention. Recent estimates for their physiological oxygen requirements are very low (Sperling *et al.,* 2013a; Mills *et al.,* 2014)—mostly well below the traditional 1% to 10% PAL atmospheric O_2_ hypothesized for the hundreds of millions of years leading up to the arrival of animals. This possibility has contributed to the idea that rising O_2_ played little, if any, role in triggering the beginnings of animal life (Mills and Canfield, 2014; Mills *et al.,* 2014; Butterfield, 2018; Hammarlund *et al.,* 2018; Mills *et al.,* 2018, reviewed in Cole *et al.,* 2020). Much of this discussion has centered on animal physiology—that is, the often very low thresholds of O_2_ tolerance for animals in modern natural and experimental settings (Sperling *et al.,* 2013a; Mills *et al.,* 2014)—and whether or not the boring billion interval could have supported animal life. If one argues that there was ample O_2_, then their later appearance, roughly a billion years after the first eukaryotes, could well have little or nothing to do with changes in environmental oxygen levels.

Alternatively, we suggest that those early redox conditions, because of their dynamics; heterogeneities; and the potential, on average, for low levels of O_2_ in the surface ocean could have combined to form a challenging ecological and evolutionary landscape for the earliest animal life. It remains possible that animals could have emerged and persisted under such conditions, but in the absence of a record it is hard to know. Rather than focusing on the origin of animals, we instead have directed our attention to times when the environment and life show dramatic, easily recognized in-phase changes—and whether the former drove the latter. The coincidences are strong, and it follows reasonably that ramped-up eukaryotic life and the molecular clock and geochemical suggestions for animals at roughly 800 to 700 Ma could reflect increasing and more stable O_2_ in the surface oceans. Comparative studies of development, however, indicate that the earliest animals likely possessed relatively few cell types and lacked complex developmental patterning (Davidson and Erwin, 2006, 2009; Erwin, 2020), with more complex development and morphology arising convergently in different lineages during the Ediacaran. Such low complexity for early metazoans relative to those that appeared later is consistent with still challenging environmental conditions in the late Tonian and Cryogenian and the possibility of higher (perhaps episodically) O_2_ during the Ediacaran.

Given the temporal coincidence for records of biotic and environmental change, the dissenting opinion to oxygen's chokehold on evolutionary development is that eukaryotic expansion and the appearance of animals must have *driven* oxygenation—giving us another chicken-or-the-egg argument. The notion that the rise of complex life and its profound ecological impacts was a cause rather than consequence of oxygenation has been a popular view (Lenton *et al.,* 2014, reviewed in Erwin *et al.,* 2011). We acknowledge complex life's potential role in facilitating further oxygenation via a set of positive feedbacks, but perhaps less convincing is the idea that early algae and animals were responsible for the NOE, including the signals centered around 800 Ma (compare Lenton *et al.,* 2014). For example, any assertion that planktonic grazing by large eukaryotes might have sufficiently enhanced the biological pump by increasing particle size and thus settling rates, with consequent increases in organic burial efficiency, must acknowledge that the advent of such contributions is not well dated and that grazing also disaggregates particles. Any contribution from fecal pellet production (Logan *et al.,* 1995) certainly came much later and may have been triggered by increasing O_2_. Lastly, the possibility that early, minute sponges, through filtering, stripped large amounts of reduced C and thus O_2_ sinks from the water column demands operation at a massive scale. Currently, there is no evidence in the record for the predicted voluminous burial of sponge biomass.

Rather than stimulating oxygenation, animals can also trigger negative feedbacks through bioturbation, which can decrease the O_2_ content of the atmosphere through enhanced sulfide and organic matter oxidation and phosphorus retention in the sediments (Canfield and Farquhar, 2009; Boyle *et al.,* 2014; Lenton *et al.,* 2014). In any case, this hypothesis is largely irrelevant to our discussion, as biological mixing of sediments on marine shelves at the levels required to impact global-scale biogeochemical cycles was delayed well into the Paleozoic (likely at least the late Silurian)—hundreds of millions of years after our ∼800 Ma window (Tarhan *et al.,* 2015). There is no doubt that these and perhaps many other processes related to complex life contributed to the pattern of oxygenation, particularly as positive and perhaps negative feedbacks, but it is far less clear to us that these influences were more important than extrinsic factors, such as tectonics.

We imagine a boring billion that was permissive of eukaryotic development, which emerged under challenging conditions with or without subtle environmental drivers. Certainly O_2_ oases as early footholds in a world rife with O_2_ heterogeneity during the Archean (Olson *et al.,* 2013) and the Proterozoic (Reinhard *et al.,* 2016) could have played a role, perhaps allowing emergence without major spikes in diversification and proliferation for aerobic prokaryotes and eukaryotes. Only later, however, did vast radiation of eukaryotes and complex eukaryote-dominated ecosystems follow—coincident with, and perhaps in response to, major environmental change. Pitched this way, the end-member proponents for and against the need for rising O_2_ as a prerequisite for evolution of complex life find a point of compromise. Further, molecular and geochemical evidence point to initial animal divergence perhaps one to two hundred million years before the rich body fossil record, suggesting a macroevolutionary lag that separated the initial developmental toolkits and much later ecological success (Erwin *et al.,* 2011) perhaps also triggered by environmental O_2_ and/or nutrient abundance (Evans *et al.,* 2018). Our goal here has been only to touch upon highlights of the oxygen-animal debate with the hope that the reader will leave feeling curious and wanting more information on this lively and important conversation. For additional reading, Cole *et al.* (2020) provided a thorough discussion addressing all sides of the story in rigorous detail.

To speculate more broadly, questions about the importance of oxygen in eukaryotic evolution remain central, fostered by debates specific to animals but extending to early eukaryotes more generally, including suggestions that the earliest eukaryotes might have required very low O_2_ or been anaerobic (Mentel and Martin, 2008; Porter *et al.,* 2018). The argument for oxygen pacing the rise of eukaryotes to ecological dominance starts from a single assumption—namely, that it is energetically favorable, in a net sense, for a eukaryote to live in a persistently oxic environment. This argument makes sense intuitively, as the energy yield from aerobic respiration is significantly greater than that of any anaerobic pathway. However, living in an oxic environment also comes at great expense. An enormous amount of energy production at the cellular level is devoted to maintenance, as cells continuously battle against damage of biomolecules from a variety of processes (racemization, cross-linking, depurination, oxidation, etc.). Many of these destructive processes occur more rapidly in oxic environments, and the initial cost of monomer synthesis is also higher (reviewed in Lever *et al.,* 2015). Altogether, this relationship amounts to an energy requirement that is over an order of magnitude greater for a cell to survive over time in an oxic environment—a number that approaches or exceeds the increased ATP yield from aerobic oxidation of glucose.

If the initial energetic cost/benefit were not positive, evolution would not necessarily have exploited oxygenated environments in the mid-Proterozoic for that reason, particularly if anoxic habitat space were abundant in the photic zone. As the surface oceans became more pervasively oxygenated, however, anoxic habitats would have correspondingly declined, potentially providing the evolutionary pressure to develop adaptations that allowed for life under the extreme conditions of persistently oxygenated waters—leading ultimately to innovation and correspondingly greater diversity among eukaryotes, including the very energetic processes of subsequent animal life.

This alternative argument, based more on shifting habit availability than energy yield, provides a possible explanation for why eukaryotes including animals responded to rising oxygen in the late Neoproterozoic rather than the weakly oxygenated mid-Proterozoic surface oceans, even if the O_2_ concentrations were sufficient to support basal animal life. In other words, the initial steps toward well-oxygenated settings were motivated less by cellular oxygen need and associated energy benefits and more a response to shallow anaerobic and aerobic environments that were declining and expanding, respectively.

## 7. What Came Next?—The Fabric of Latest Proterozoic Oxygenation

One model is that oxygen rose in the atmosphere about 800 Ma to a new baseline—perhaps well above the average levels of the boring billion—but that it stayed still mostly lower than today through the remainder of the Proterozoic and into the Paleozoic, which started at 541 Ma. An issue of particular interest is whether O_2_ in the atmosphere and oceans continued to be dynamic, rising and falling above the new baseline that loosely defined the NOE (Fig. 3). In addition to the indicators for significant oxygenation centered around 800 Ma, some evidence suggests increasing O_2_ in the immediate wake of Marinoan glaciation about 635–630 Ma (Sahoo *et al.,* 2012, 2016; Kunzmann *et al.,* 2017, compare Miller *et al.,* 2017). One implication is that O_2_ in the atmosphere and oceans may have dipped following the rise at roughly 800 Ma, perhaps before the onset of glaciation, and stayed relatively low or variable during the Cryogenian and then rose again (Cheng *et al.,* 2018).

Some recent organic geochemical data suggest that bacteria continued to dominate primary production until the interglacial between the Sturtian and Marinoan glaciations (Brocks *et al.,* 2017) starting at roughly 660 Ma, while other data indicate a proliferation of eukaryotes starting closer to 800–750 Ma (Brocks *et al.,* 2017; Brocks, 2018; Isson *et al.,* 2018). The interglacial is the same interval that produced the earliest records of animals (sponges; Love *et al.,* 2009; Love and Summons, 2015), as well as diversification among eukaryotes more generally, including emergence of the earliest predatory rhizarians (Brocks *et al.,* 2017)—a group that includes radiolaria and foraminifera.

The microfossil data point to a Tonian rise in eukaryotic diversity, followed by a decline before and extending through the Cryogenian, and a large jump in diversity in the early Ediacaran (Cohen and Macdonald, 2015; Riedman and Sadler, 2018; Fig. 1). The microfossils could thus be taken to track a rise in O_2_ in the atmosphere and oceans around 800 Ma followed by a pre-Sturtian drop and post-Marinoan bounce back up (Fig. 1)—again, the latter possibly initiated at, and triggered by, the end of glaciation (Planavsky *et al.,* 2010; Sahoo *et al.,* 2012).

It now seems clear from the records of environmental and biological change that the period of particular interest spans from just before 800 Ma though the Cryogenian (720–635 Ma), defined by snowball Earth glaciations, and into the Ediacaran. This timeline raises questions about the timing, structure, and duration of the conjectured oxygenation event (Fig. 3), including the possibility of rising and falling biospheric O_2_ in this window—leading to both opportunities and challenges for innovation among complex organisms. It also leaves open the possibility of cause-and-effect relationships between oxygenation and global-scale glaciation.

Other data suggest a persistence of mostly low O_2_ at least in the deep oceans throughout the Ediacaran (Canfield *et al.,* 2008; Sperling *et al.,* 2015a)—perhaps in combination with transient increases in the atmosphere and oceans (Kendall *et al.,* 2015; Sahoo *et al.,* 2016; see also Shields *et al.,* 2019; compare Ostrander *et al.,* 2020, and Jin *et al.,* 2021). This fabric of latest Proterozoic O_2_ and specifically ephemeral oceanic oxygenation may have played a role in early animal evolution and Ediacaran carbon cycling, and the interval from 580 to 550 Ma containing the famous Shuram carbon isotope anomaly (Grotzinger *et al.,* 2011) has emerged as a time of particular interest (*e.g.,* Fike *et al.,* 2006; Canfield *et al.,* 2007; McFadden *et al.,* 2008; Hardisty *et al.,* 2017; Li *et al.,* 2017a; Evans *et al.,* 2018; Shi *et al.,* 2018; Rooney *et al.,* 2020). Perhaps both innovation and extinction among the early animals were driven by ephemeral environmental change (Evans *et al.,* 2018; Muscente *et al.,* 2019).

The natural corollary in this discussion is that biospheric O_2_ must have risen to something like modern levels much later (Dahl *et al.,* 2010; Lu *et al.,* 2018), particularly in the deep ocean (Stolper and Keller, 2018), and that pulsed oxygenation in an ocean that was still mostly anoxic may have continued even into the Paleozoic. From this vantage, the NOE becomes a protracted and dynamic event, much like the GOE (as reviewed in Lyons *et al.,* 2014). It follows that the ensuing “Cambrian explosion” of animal life and its possible relationship to environmental change, including dramatic and persistent shifts in atmospheric and oceanic O_2_ and stably oxic conditions in the surface-most waters, remains unresolved (Sperling *et al.,* 2013b, 2016; Chen *et al.,* 2015; Jin *et al.,* 2016; Li *et al.,* 2017b, 2018; Lu *et al.,* 2018). Nonetheless, we suggest that the oceans overall remained dynamic, heterogeneous, broadly reducing, and transitional—much like the Proterozoic—well beyond the end of the Ediacaran.

The latter part of the Ediacaran witnessed impressive diversification among the animals and corresponding ecosystem complexity, including the emergence of bilaterians, large mostly soft body plans, the earliest infaunal activity, mobility, advances in heterotrophy, possible stem-group molluscs, and even the first skeletons (reviewed in Droser *et al.,* 2017). These steps can be viewed as energy-demanding innovations favored by higher O_2_ availability. The tremendous advantages of O_2_-dependent metabolisms are reviewed in the work of Taverne *et al.* (2018). On the other hand, some researchers have argued that because O_2_ requirements for animals can be very low (Sperling *et al.,* 2013a, 2015b), oxygenation events may not have been necessary to explain the beginnings of animals or even bilaterians.

Questions remain as to whether often-cited modern systems and organisms, capturing animal life at its lowest levels of tolerance, are meaningful analogs for the ecological complexity and possible O_2_-demanding taxa of the late Ediacaran, including, for example, the mobility of *Kimberella* (a putative stem group mollusc) as motivated by grazing (Sperling *et al.,* 2015b). We acknowledge that many of the Ediacaran animals may have been happy even at very low O_2_ (Sperling *et al.,* 2015b, compare Evans *et al.,* 2018), but absent major increases in oxygenation, benthic conditions—even during the Ediacaran—could have been frequently inhospitable. Further, as complexity of both body plans and behaviors increased, so too would have O_2_ demand. If it turns out that major events in eukaryote diversification, the rise of animals, and even the patterns of Ediacaran innovation do indeed line up with independent indicators of rising and perhaps pulsed oxygenation (*e.g.,* Sahoo *et al.,* 2016), future research will continue to explore possible cause-and-effect relationships. At this point, however, it is critical to remember that animals need oxygen. While the earliest animals may have had very low oxygen requirements and perhaps even evolved in a low O_2_ environment, it seems equally important to acknowledge the trajectory of their subsequent development and evolution—which was toward more energetically demanding body plans and behaviors, requiring higher and more stable environmental O_2_. Even if sufficient O_2_ existed for some sponge species in the mid-Proterozoic, it does not mean that all animals would have been happy living at that time. At some point, by any estimation, environmental O_2_ began to matter. The affinity animals have for oxygenated waters is apparent today and played out countless times in the geological record (*e.g.,* Hammarlund *et al.,* 2017).

## 8. Summary and Implications beyond Earth

We can think of Precambrian oxygenation as occurring in steps with continued variability around increasing baseline *p*O_2_, including the possibility of rising and falling oxygen through the boring billion, the Ediacaran, and into the Paleozoic (Fig. 3; Li *et al.,* 2017b)—this variability may have driven extinction and innovation in first-order ways. This conceptual model, of course, must be weighed first and foremost against the data, along with a better marriage of those results with numerical models and large-scale geological observations. The grand challenge is to link the environmental and tectonic patterns extracted from the rock record for the oceans, atmosphere, and continents to the parallel patterns in the evolution of Earth's biosphere. Such an effort will ultimately need to focus on the least constrained portion of the ocean—the surface waters particularly along ocean margins, where all or most of the action was for complex life. The most important message to carry forward from the fossil record is that the numbers game can be misleading, given the myriad intrinsic biases and gaps in the data available; we need better community agreement on how to assess patterns of diversity and ecological innovation. This fabric is something we need to test and refine, including the timing of the “terminal oxygenation”—that is, the jump to dominantly strongly oxygenated conditions perhaps long after the end of the Proterozoic (*e.g.,* Bergman *et al.,* 2004; Dahl *et al.,* 2010).

While details remain poorly known, there is general agreement that O_2_ levels in the atmosphere and oceans during Earth's middle history were likely low compared to the intervals before and after. This possibility carries important implications for life, but it also impacts our search for life on distant exoplanets. Specifically, the atmospheric estimates on the low end (perhaps much less than 1% of PAL) suggest that O_2_ in the atmosphere may have been below detection were distant observers scanning our atmosphere for signs of life during the boring billion (Reinhard *et al.,* 2017b)—despite oceans with long-established prokaryotic life and emerging eukaryotic diversity. Such conditions might require observing indirect “proxies” for O_2_, such as O_3_ (Reinhard *et al.,* 2019). As discussed, these muted levels of O_2_ might have reflected correspondingly low levels of primary production in the oceans.

There is another noteworthy consequence of such low O_2_ levels. We can imagine oceans during the boring billion with appreciable levels of dissolved sulfate (*e.g.,* Kah *et al.,* 2004; Fakhraee *et al.,* 2019; Blättler *et al.,* 2020). In contrast to the preceding Archean eon, when marine sulfate concentrations were vanishingly small (Habicht *et al.,* 2002; Crowe *et al.,* 2014) and methane (CH_4_) undoubtedly contributed significantly to greenhouse warming, both methane production and preservation may have been challenged during the boring billion. Appreciable sulfate can limit microbiological methanogenesis because of the competition with sulfate-reducing microbes for organic substrates. Sulfate can also favor significant loss of methane in marine sediments and seawater via anaerobic oxidation of methane. The low levels of methane sourced to the atmosphere could then have faced considerable photochemical destruction if O_2_ and thus ozone (O_3_) were very low. It is therefore possible that both O_2_ and CH_4_—both promising biosignatures in exoplanet research—would have been below remote detection limits. Moreover, the detection of both, a classic disequilibrium biosignature (*e.g.,* Sagan *et al.,* 1993), would have been lost to observers. Such co-occurrences, because of the incompatibilities of reduced and oxidized gases, are commonly attributed to appreciable biological production of both. The possibility of Earth's middle history failing to reveal life to remote observers through the presence of O_2_ and CH_4_ alone or in combination has helped fuel the concept of a false negative—that is, “cryptic biospheres that are widespread and active on a planet's surface but are ultimately undetectable or difficult to detect in the composition of a planet's atmosphere” (Reinhard *et al.,* 2017b). Importantly, as part of the false-negative concept, we can also imagine oases of high O_2_ in the surface oceans that supported aerobic life beneath an O_2_-poor atmosphere.

Earth's history, therefore, has taught us that missed signatures of life may be just as important to consider as false positives in searches beyond our solar system. This example is only one highlight on the long list of many reasons to include studies of Earth's past when developing a toolbox for exploring distant habitability and life. More generally, this discussion of ancient Earth is filled with many other examples of how a complex, evolving planetary system can sustain habitability and life, and those lessons must be considered when assessing the habitability and prospects for life detection on distant worlds.

## Supplementary Material

Supplemental data

## Notes

Associate Editor: Christopher McKay

## Abbreviations Used

Ga: billion years ago
GOE: Great Oxidation Event
LIPs: large igneous provinces
Ma: million years ago
NOE: Neoproterozoic Oxidation Event
PAL: present atmospheric level
