# Supplementary material for: Oxygenation, Life, and the Planetary System during Earth's Middle History: An Overview
Source: Astrobiology. 2021 Aug 16;21(8):906–23. doi: 10.1089/ast.2020.2418 (PMC8403206; doi:10.1089/ast.2020.2418)
Supplement: Supplemental data [file Supp_Data.pdf]

Supplementary Information:

Figure S1. Annotated version of Figure 1 and accompanying table. References for evolutionary milestones are listed at the bottom of the table.

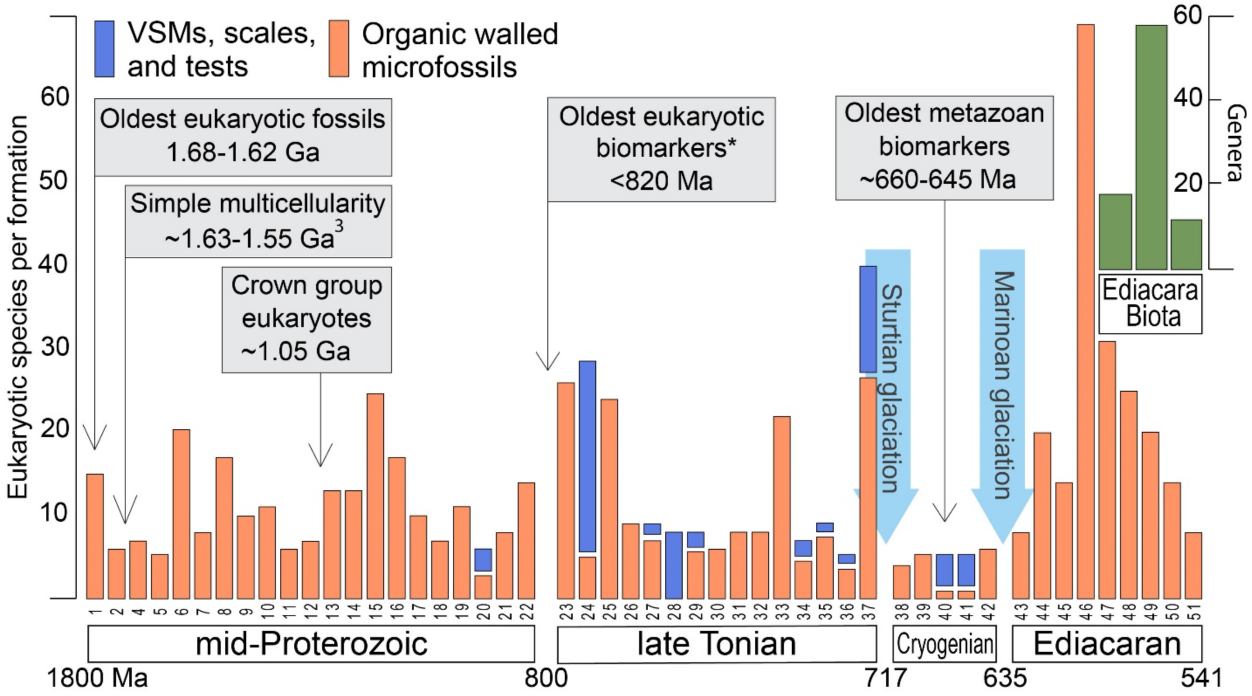

No.	Formation or Group	Approximate Age (Ma)	Eukaryotic Diversity	Primary References
1	Changzhougou Formation	1650	15	Lamb et al., 2009; Zhang, 1997
2	Chengcheng Formation	1650	6	Miao et al., 2019
3	Tuanshanzi Formation	1630	3	Qu et al., 2018
4	Ruyang Group	1645	7	Yin et al., 2005; Agić et al., 2017
5	Gaoyuzhuang Formation	1560	5	Shi et al., 2017
6	Beidajian Formation	1500	20	Agić et al., 2017
7	Roper Group	1455	8	Javaux et al., 2004; Javaux and Knoll, 2017
8	Kaltasy Formation	1435	17	Sergeev et al., 2016
9	Thule Supergroup	1135	10	Samuelsson et al., 1999
10	Atar/El Mariti Group	1100	11	Beghin et al., 2017
11	Nonesuch Shale	1095	6	Strother et al., 2011
12	Olenek Uplift	1050	7	Stanevich et al., 2009
13	Torridonian Succession	1025	13	Battison and Brasier, 2012; Strother et al., 2012
14	Lakhanda Group	1025	13	Jankauskas, 1989
15	Shaler Group	~1000	25	Loron and Moczydłowska, 2018
16	Tongjiazhuang Formation	~1000	17	Li et al., 2019

17	Mbuji-Mayi Supergroup	975	10	Baludikay et al., 2016
18	Halkal / Hulkal Formation	895	7	Sharma and Shukla, 2012; Maithy and Babu, 1996
19	Liulaobei Formation	850	11	Tang et al., 2013
20	Huainan Group	850	6	Xiao et al., 2014a
21	Basinal Assemblage of Little Dal Group	>820	8	Hofmann, 1985
22	Wynniatt Formation	>810	14	Hofmann and Rainbird, 1994; Butterfield and Rainbird, 1998
23	Alinya Formation	800	26	Zang, 1995; Riedman and Porter, 2016
24	Fifteenmile Group	800	28	Allison and Hilgert, 1986; Allison and Awramik, 1989; Cohen and Knoll, 2012
25	Svanbergfjellet Formation	800	24	Butterfield et al., 1994
26	Hunnberg Formation	790	9	Knoll, 1984
27	Chichikan Formation	785	9	Sergeev and Schopf, 2010
28	Urucum Formation	766	8	Morais et al., 2017
29	Draken Formation	765	8	Knoll et al., 1991
30	Backlundtoppen Formation	765	6	Knoll et al., 1989
31	Batsfjord Formation	760	8	Vidal and Siedlecka, 1983
32	Visingsö Group (Lower and Middle)	760	8	Vidal, 1976
33	Visingsö Group (upper)	750	22	Loren and Moczydłowska, 2018; Vidal, 1976
34	Rysso Formation	745	7	Knoll and Calder, 1983
35	Uinta Mountain Group	740	9	Dehler et al., 2010
36	Pahrump Group, Death Valley	740	5	Corsetti et al., 2003
37	Chuar Group	740	39	Vidal and Ford, 1985; Nagy et al., 2007; Porter et al., 2003; Porter, 2016
38	Areyonga Formation	660	4	Riedman et al., 2014
39	Aralka Formation	660	5	Riedman et al., 2014
40	Rasthof Formation	660	5	Bosak et al., 2011a; Dalton et al., 2013
41	Tayshir Formation	660	5	Bosak et al., 2011b
42	Tapley Hill Formation	650	6	Riedman et al., 2014
43	Lantian Formation	<635	8	Xunlai et al., 1999; Yuan et al., 2001
44	Ura Formation	<635	20	Sergeev et al., 2011
45	Parsha Formation	<635	14	Golubkova et al., 2010
46	Doushantuo Formation	635-580	69	Xiao et al., 2002; Xiao et al., 2004; Xiao et al., 2014b
47	Dey-Dey Formation-Officer Basin	580-560	31	Grey, 2005
48	Dey-Dey Formation-Amadaeus Basin	580-560	25	Zang and Walter, 1992
49	Kelt'ma Assemblage	580-552	20	Vorob'eva et al., 2009
50	Redkino Suite	550	14	Burzin et al., 1996

51	Nama Group	550	8	Germs et al., 1986; Cohen et al., 2009
	Oldest eukaryotic microfossils	1680-1620		Knoll and Nowak, 2017; Javaux and Lepot, 2018
	Simple multicellularity	1630-1550		Zhu et al., 2016
	Crown group eukaryotes	1.05		Butterfield, 2000; Gibson et al., 2017; but see also Bengtson et al., 2017
	Oldest eukaryotic biomarkers	~800		Brocks et al., 2017
	Oldest metazoan biomarkers	~660-645		Love et al., 2009; Love and Summons, 2015

Figure S2. Annotated version of Figure 3 with accompanying table.

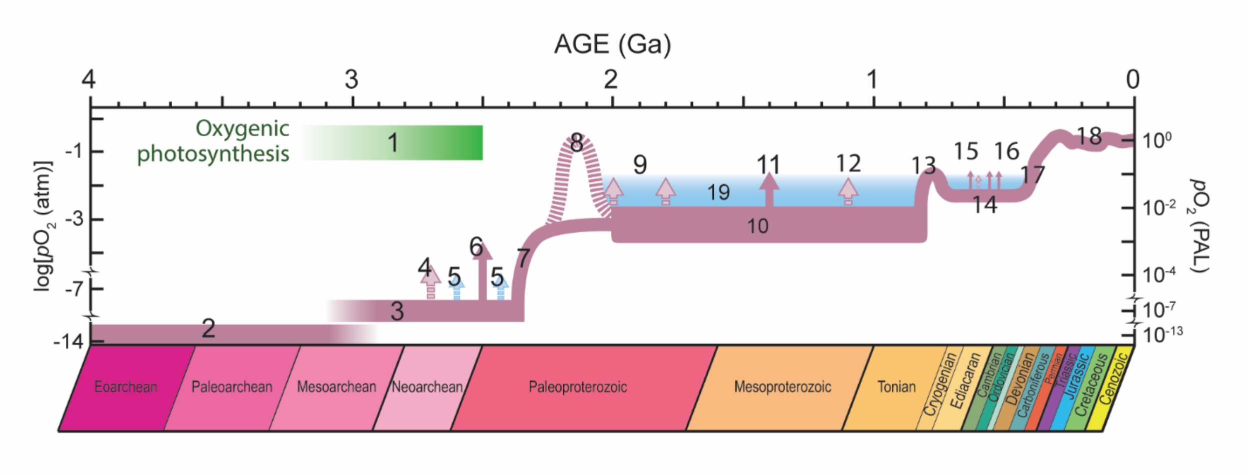

No.	Explanation	References
1	The evolution of oxygenic photosynthesis occurred prior to the end of the Archaean.	Planavsky et al., 2014a; Satkoski et al., 2015
2	Prior to the evolution of oxygenic photosynthesis, O_2 production restricted to photolytic reactions in the upper atmosphere involving CO_2 and H_2O.	Kasting and Walker, 1981
3	After the evolution of oxygenic photosynthesis, dynamic models suggest system stability ~10^{-7} PAL.	Pavlov and Kasting, 2002; Goldblatt et al., 2006
4	Possible 'whiff' of O_2 or oxygen oasis suggested from Jeerinah Formation, Western Australia.	Scott et al., 2011
5	Hypothetical oscillations predicted to accompany the GOE transition because of low buffering capacity of the system nearing conditions that favored change in steady state.	
6	Confident 'whiff' of O_2 prior to the GOE recorded in the Mt. McRae Shale, Western Australia.	Anbar et al., 2007
7	'Great Oxidation Event' dated to ~2.33 Ga by the disappearance of non-mass-dependent fractionation of sulfur observed in pyrite.	Luo et al., 2016
8	Potential O_2 'overshoot' suggested by interpretation of the Lomagundi Event (~200 Myr interval of extremely ^{13}C enriched carbonate rocks) as the product of enhanced burial of oxygenic-photosynthetically derived organic carbon; significant uncertainty surrounds this interpretation, including the possibility that O_2 gained passed only transiently through the atmosphere and accumulated rather as elevated oxidant availability in the oceans (e.g., sulfate). Also poorly known are the controls on declining O_2/oxidants at the end of the Lomagundi Event.	Bekker and Holland, 2012; cf., Krissansen-Totton et al., 2015; Blättler et al., 2018; Ossa Ossa et al., 2018
9	Potential transient oxygenation events in the later	Planavsky et al., 2018; Mänd et al., 2020

	Paleoproterozoic (after the falling limb of the Lomagundi carbon isotope excursion at ~2.0 and 1.8 Ga)	
10	Low atmospheric O_2 levels (0.1-1% PAL based on the δ^{53}Cr record in iron formations and shales) characterized the bulk of the mid-Proterozoic.	Planavsky et al., 2014b; Cole et al., 2016
11	Multiple lines of evidence point to a transient increase in O_2 ~1.4 Ga.	Zhang et al., 2016; Liu et al., 2016; Hardisty et al., 2017; Yang et al., 2017; Diamond et al., 2018
12	The δ^{53}Cr record of carbonates suggests the possibility of transient oxygenation ~1.1 Ga.	Gilleaudeau et al., 2016
13	The δ^{53}Cr record of iron formations, iron stones, and organic rich shales shows significant fractionations beginning ~800 Ma, indicating the onset of oxidative Cr cycling in terrestrial weathering environments.	Planavsky et al., 2014; Cole et al., 2016
14	Multiple lines of evidence indicate that widespread anoxic conditions persisted through the bulk of the late Neoproterozoic and early Palaeozoic.	Sperling et al., 2015; Wallace et al., 2017
15	Transient spikes in redox-sensitive trace metal concentrations in the Doushantuo Formation indicate transient reductions in spatial extent of anoxic marine conditions.	Sahoo et al., 2016; Shi et al., 2018
16	Multiple studies have suggested oxygenation in the Middle Cambrian (~521 Ma) on the basis of various proxies applied to strata of south China, other work has shown a subsequent continuation of widespread anoxia, indicating that this event was likely transient if genuine.	Chen et al., 2015; Jin et al., 2017; Li et al., 2017
17	A stepwise increase to near modern pO_2 is suggested to have accompanied the rise of vascular land plants; multiple lines of evidence support this notion.	Dahl et al., 2010; Lenton et al., 2016; Wallace et al., 2017
18	Remainder of pO_2 curve adapted from GEOCARBSULF, which is based on the δ^{13}C record of marine carbonates.	Berner, 2006: Schachat et al., 2018
19	Some model predictions suggest a lack of stability in the pO_2 range consistent with the δ^{53}Cr record and instead argue for higher (1-10% PAL) pO_2 throughout the mid-Proterozoic.	Daines et al., 2017

Supplementary References:

Agić, H., Moczydłowska, M., and Yin, L. (2017) Diversity of organic-walled microfossils from the early Mesoproterozoic Ruyang Group, North China Craton–A window into the early eukaryote evolution. *Precambrian Res* 297:101-130.

Allison, C.W. and Awramik, S.M. (1998) Organic-walled microfossils from earliest Cambrian or latest Proterozoic Tindir Group rocks, northwest Canada. *Precambrian Res* 43:253-294.

Allison, C.W. and Hilgert, J.W. (1986) Scale microfossils from the Early Cambrian of northwest Canada. *J Paleontol* 60:973-1015.

Anbar, A.D., Duan, Y., Lyons, T.W., Arnold, G.L., Kendall, B., Creaser, R.A., Kaufman, A.J., Gordon, G.W., Scott, C., Garvin, J., and Buick, R. (2007) A whiff of oxygen before the great oxidation event? *Science* 317:1903-1906.

Baludikay, B.K., Storme, J.Y., François, C., Baudet, D., and Javaux, E.J. (2016) A diverse and exquisitely preserved organic-walled microfossil assemblage from the Meso–Neoproterozoic Mbuji-Mayi Supergroup (Democratic Republic of Congo) and implications for Proterozoic biostratigraphy. *Precambrian Res* 281**:**166-184.

Battison, L. and Brasier, M.D. (2012) Remarkably preserved prokaryote and eukaryote microfossils within 1 Ga-old lake phosphates of the Torridon Group, NW Scotland. *Precambrian Res* 196:204-217.

Bekker, A. and Holland, H.D. (2012) Oxygen overshoot and recovery during the early Paleoproterozoic. *Earth Planet Sci Lett* 317:295-304.

Beghin, J., Storme, J.Y., Blanpied, C., Gueneli, N., Brocks, J.J., Poulton, S.W., and Javaux, E.J. (2017) Microfossils from the late Mesoproterozoic–early Neoproterozoic Atar/el Mreïti group, Taoudeni basin, Mauritania, northwestern Africa. *Precambrian Res* 291:63-82.

Bengtson, S., Sallstedt, T., Belivanova, V., and Whitehouse, M. (2017) Three-dimensional preservation of cellular and subcellular structures suggests 1.6 billion-year-old crown-group red algae. *PLoS Biol* 15:e2000735.

Berner, R. A. (2006) GEOCARBSULF: a combined model for Phanerozoic atmospheric O_2 and CO_2. *Geochim Cosmochim Acta* 70:5653-5664.

Blättler, C.L., Claire, M.W., Prave, A.R., Kirsimäe, K., Higgins, J.A., Medvedev, P.V., Romashkin, A.E., Rychanchik, D.V., Zerkle, A.L., Paiste, K., and Kreitsmann, T. (2018) Two-billion-year-old evaporites capture Earth's great oxidation. *Science* 360:320-323.

Bosak, T., Lahr, D.J.G., Pruss, S.B., Macdonald, F.A., Dalton, L., and Matys, E. (2011a) Agglutinated tests in post-Sturtian cap carbonates of Namibia and Mongolia. *Earth Planet Sci Lett* 308:29-40.

Bosak, T., Macdonald, F., Lahr, D., and Matys, E. (2011b) Putative Cryogenian ciliates from Mongolia. *Geology* 39:1123-1126.

Brocks, J.J., Jarrett, A.J., Sirantoine, E., Hallmann, C., Hoshino, Y., and Liyanage, T. (2017) The rise of algae in Cryogenian oceans and the emergence of animals. *Nature* 548:578-581.

Burzin, M.B. (1996) Late Vendian (Neoproterozoic III) microbial and algal communities of the Russian Platform: models of facies-dependent distribution, evolution and reflection of basin development. *Rivista Italiana di Paleontologia e Stratigrafia (Research In Paleontology and Stratigraphy)* 102.

Butterfield, N.J. (2000) Bangiomorpha pubescens n. gen., n. sp.: implications for the evolution of sex, multicellularity, and the Mesoproterozoic/Neoproterozoic radiation of eukaryotes. *Paleobiology* 26:386-404.

Butterfield, N.J., Knoll, A.H., and Swett, K. (1994) Paleobiology of the Neoproterozoic Svanbergfjellet Formation, Spitsbergen. *Lethaia* 27:76-76.

Butterfield, N.J. and Rainbird, R.H. (1998) Diverse organic-walled fossils, including "possible dinoflagellates," from the early Neoproterozoic of arctic Canada. *Geology* **26**:963-966.

Chen, X., Ling, H.F., Vance, D., Shields-Zhou, G.A., Zhu, M., Poulton, S.W., Och, L.M., Jiang, S.Y., Li, D., Cremonese, L., and Archer, C. (2015) Rise to modern levels of ocean oxygenation coincided with the Cambrian radiation of animals. *Nature Comm* 6:1-7.

Cohen, P.A., Bradley, A., Knoll, A.H., Grotzinger, J.P., Jensen, S., Abelson, J., Hand, K., Love, G., Metz, J., McLoughlin, N., and Meister, P. (2009) Tubular compression fossils from the Ediacaran Nama group, Namibia. *J Paleontol* 83:110-122.

Cohen, P.A. and Knoll, A.H. (2012) Scale microfossils from the mid-Neoproterozoic Fifteenmile Group, Yukon Territory. *J Paleontol* **86**:775-800.

Cole, D.B., Reinhard, C.T., Wang, X., Gueguen, B., Halverson, G.P., Gibson, T., Hodgskiss, M.S., McKenzie, N.R., Lyons, T.W., and Planavsky, N.J. (2016) A shale-hosted Cr isotope record of low atmospheric oxygen during the Proterozoic. *Geology* 44:555-558.

Corsetti, F.A., Awramik, S.M., and Pierce, D.A. (2003) Complex microbiota from snowball Earth times: microfossils from the Neoproterozoic Kingston Peak Formation, Death Valley, USA. *Proc Natl Acad Sci USA* 100:4399-4404.

Dahl, T.W., Hammarlund, E.U., Anbar, A.D., Bond, D.P., Gill, B.C., Gordon, G.W., Knoll, A.H., Nielsen, A.T., Schovsbo, N.H., and Canfield, D.E. (2010) Devonian rise in atmospheric oxygen correlated to the radiations of terrestrial plants and large predatory fish. *Proc Natl Acad Sci USA* 107:17911-17915.

Daines, S.J., Mills, B.J., and Lenton, T.M. (2017) Atmospheric oxygen regulation at low Proterozoic levels by incomplete oxidative weathering of sedimentary organic carbon. *Nature Comm* 8:14379.

Dehler, C. M., Fanning, C. M., Link, P. K., Kingsbury, E. M., and Rybczynski, D. (2010) Maximum depositional age and provenance of the Uinta Mountain Group and Big Cottonwood Formation, northern Utah: Paleogeography of rifting western Laurentia. *Geol Soc Am Bull* 122:1686-1699.

Diamond, C.W., Planavsky, N.J., Wang, C., and Lyons, T.W. (2018) What the ~1.4 Ga Xiamaling Formation can and cannot tell us about the mid-Proterozoic ocean. *Geobiology* 16:219-236.

Germs, G.J., Knoll, A.H., and Vidal, G. (1986) Latest Proterozoic microfossils from the Nama Group, Namibia (South West Africa). *Precambrian Res* 32:45-62.

Gibson, T.M., Shih, P.M., Cumming, V.M., Fischer, W.W., Crockford, P.W., Hodgskiss, M.S., Wörndle, S., Creaser, R.A., Rainbird, R.H., Skulski, T.M., and Halverson, G.P. (2018) Precise age of Bangiomorpha pubescens dates the origin of eukaryotic photosynthesis. *Geology* 46:135-138.

Gilleaudeau, G.J., Frei, R., Kaufman, A.J., Kah, L.C., Azmy, K., Bartley, J.K., Chernyavskiy, P., and Knoll, A.H. (2016) Oxygenation of the mid-Proterozoic atmosphere: clues from chromium isotopes in carbonates. *Geochem Perspect Lett* 2:178-187.

Goldblatt, C., Lenton, T.M., and Watson, A.J. (2006) Bistability of atmospheric oxygen and the Great Oxidation. *Nature* 443:683.

Golubkova, E.Y., Raevskaya, E.G., and Kuznetsov, A.B. (2010) Lower Vendian microfossil assemblages of East Siberia: significance for solving regional stratigraphic problems. *Strat Geol Corr* 18:353-375.

Grey, K. *Ediacaran palynology of Australia.* 31, (Association of Australasian Palaeontologists, 2005).

Hardisty, D.S., Lu, Z., Bekker, A., Diamond, C.W., Gill, B.C., Jiang, G., Kah, L.C., Knoll, A.H., Loyd, S.J., Osburn, M.R., and Planavsky, N.J. (2017) Perspectives on Proterozoic surface ocean redox from iodine contents in ancient and recent carbonate. *Earth Planet Sci Lett* 463:159-170.

Hofmann, H.J. (1985) The mid-Proterozoic Little Dal macrobiota, Mackenzie Mountains, north-west Canada. *Palaeontol* 28:331-354.

Hofmann, H. J. & Rainbird, R. H. Carbonaceous megafossils from the Neoproterozoic Shaler Supergroup of Arctic Canada. *Palaeontol* **37**, 721-732 (1994).

Jankauskas, T.V., Mikhailova, N.S., and Hermann, T.N. (1989) Mikrofossilii dokembriya SSSR [Precambrian Microfossils of the USSR]. *Trudy Instituta Geologii i Geochronologii Dokembria SSSR Akademii Nayk, Leningrad* 188.

Javaux, E.J. and Knoll, A.H., (2017) Micropaleontology of the lower Mesoproterozoic Roper Group, Australia, and implications for early eukaryotic evolution. *J Paleontol* 91:199-229.

Javaux, E.J. and Lepot, K. (2018) The Paleoproterozoic fossil record: implications for the evolution of the biosphere during Earth's middle-age. *Earth Sci Rev* 176:68-86.

Javaux, E.J., Knoll, A.H., and Walter, M.R. (2004) TEM evidence for eukaryotic diversity in mid-Proterozoic oceans. *Geobiology* 2:121-132.

Jin, C., Li, C., Algeo, T.J., Cheng, M., Lei, L., Zhang, Z., and Shi, W.E.I. (2017) Evidence for marine redox control on spatial colonization of early animals during Cambrian Age 3 (c. 521–514 Ma) in South China. *Geol Mag* 154:1360-1370.

Kasting, J.F. and Walker, J.C. (1981) Limits on oxygen concentration in the prebiological atmosphere and the rate of abiotic fixation of nitrogen. *J Geophys Res Oceans* 86:1147-1158.

Knoll, A.H. and Calder, S. (1983) Microbiotas of the late Precambrian Ryssö Formation, Nordaustlandet, Svalbard. *Palaeontol* 26:467-496.

Knoll, A.H. (1984) Microbiotas of the late Precambrian Hunnberg Formation, Nordaustlandet, Svalbard. *J Paleontol* 58:131-162.

Knoll, A.H., Swett, K., and Burkhardt, E. (1989) Paleoenvironmental distribution of microfossils and stromatolites in the Upper Proterozoic Backlundtoppen Formation, Spitsbergen. *J Paleontol* 63:129-145.

Knoll, A.H., Swett, K., and Mark, J. (1991) Paleobiology of a Neoproterozoic tidal flat/lagoonal complex: the Draken Conglomerate Formation, Spitsbergen. *J Paleontol* 65:531-570.

Knoll, A.H. and Nowak, M.A. (2017) The timetable of evolution. *Science Adv* 3:e1603076.

Krause, A.J., Mills, B.J., Zhang, S., Planavsky, N.J., Lenton, T.M., and Poulton, S.W. (2018) Stepwise oxygenation of the Paleozoic atmosphere. *Nature Comm* 9:1-10.

Krissansen-Totton, J., Buick, R., and Catling, D.C.A. (2015) Statistical analysis of the carbon isotope record from the Archean to Phanerozoic and implications for the rise of oxygen. *Am J Sci* 315:275-316.

Lamb, D.M., Awramik, S.M., Chapman, D.J., and Zhu, S. (2009) Evidence for eukaryotic diversification in the ∼1800 million-year-old Changzhougou Formation, North China. *Precambrian Res* 173:93-104.

Lenton, T.M., Dahl, T.W., Daines, S.J., Mills, B.J., Ozaki, K., Saltzman, M.R., and Porada, P. (2016) Earliest land plants created modern levels of atmospheric oxygen. *Proc Natl Acad Sci USA* 113:9704-9709.

Li, C., Jin, C., Planavsky, N.J., Algeo, T.J., Cheng, M., Yang, X., Zhao, Y., and Xie, S. (2017) Coupled oceanic oxygenation and metazoan diversification during the early–middle Cambrian? *Geology* 45*:*743-746.

Li, G., Pang, K., Chen, L., Zhou, G., Han, C., Yang, L., Wang, W., Yang, F., and Yin, L. (2019) Organic-walled microfossils from the Tonian Tongjiazhuang Formation of the Tumen Group in western Shandong, North China Craton and their biostratigraphic significance. *Gondwana Res* 76:260-289.

Liu, X.M., Kah, L.C., Knoll, A.H., Cui, H., Kaufman, A.J., Shahar, A., and Hazen, R.M. (2015) Tracing Earth's O2 evolution using Zn/Fe ratios in marine carbonates. *Geochem Perspect Lett* 2:24-34.

Loron, C. and Moczydłowska, M. (2018) Tonian (Neoproterozoic) eukaryotic and prokaryotic organic-walled microfossils from the upper Visingsö Group, Sweden. *Palynology* 42:220-254.

Loron, C.C., Rainbird, R.H., Turner, E.C., Greenman, J.W., and Javaux, E.J. (2019) Organic-walled microfossils from the late Mesoproterozoic to early Neoproterozoic lower Shaler Supergroup (Arctic Canada): diversity and biostratigraphic significance. *Precambrian Res* 321:349-374.

Love, G.D., Grosjean, E., Stalvies, C., Fike, D.A., Grotzinger, J.P., Bradley, A.S., Kelly, A.E., Bhatia, M., Meredith, W., Snape, C.E., and Bowring, S.A. (2009) Fossil steroids record the appearance of Demospongiae during the Cryogenian period. *Nature* 457:718-721.

Love, G.D. and Summons, R.E. (2015) The molecular record of Cryogenian sponges–a response to Antcliffe (2013). *Palaeontol* 58:1131-1136.

Luo, G., Ono, S., Beukes, N.J., Wang, D.T., Xie, S., and Summons, R.E. (2016) Rapid oxygenation of Earth's atmosphere 2.33 billion years ago. *Science Adv* 2:e1600134.

Maithy, P. K. and Babu, R. (1996) Carbonaceous macrofossils and organic-walled microfossils from the Halkal Formation, Bhima Group, Karnataka with remarks on age. *Palaeobotanist* 45:1-6.

Mänd, K., Lalonde, S.V., Robbins, L.J., Thoby, M., Paiste, K., Kreitsmann, T., Paiste, P., Reinhard, C.T., Romashkin, A.E., Planavsky, N.J. and Kirsimäe, K. (2020) Palaeoproterozoic oxygenated oceans following the Lomagundi–Jatuli Event. *Nature Geoscience* 13:302-306.

Miao, L., Moczydłowska, M., Zhu, S., and Zhu, M. (2019) New record of organic-walled, morphologically distinct microfossils from the late Paleoproterozoic Changcheng Group in the Yanshan Range, North China. *Precambrian Res* 321:172-198.

Morais, L., Fairchild, T.R., Lahr, D.J., Rudnitzki, I.D., Schopf, J.W., Garcia, A.K., Kudryavtsev, A.B., and Romero, G.R. (2017) Carbonaceous and siliceous Neoproterozoic vase-shaped microfossils (Urucum Formation, Brazil) and the question of early protistan biomineralization. *J Paleontol* 91:393-406.

Nagy, R.M., Porter, S.M., Dehler, C.M., and Shen, Y. (2009) Biotic turnover driven by eutrophication before the Sturtian low-latitude glaciation. *Nature Geosci* 2:415-418.

Ossa Ossa, F., Eickmann, B., Hofmann, A., Planavsky, N.J., Asael, D., Pambo, F., and Bekker, A. (2018) Two-step deoxygenation at the end of the Paleoproterozoic Lomagundi Event. *Earth Planet Sci Lett* 486:70-83.

Pavlov, A.A. and Kasting, J.F. (2002) Mass-independent fractionation of sulfur isotopes in Archean sediments: strong evidence for an anoxic Archean atmosphere. *Astrobiology* 2:27-41.

Planavsky, N.J., Asael, D., Hofmann, A., Reinhard, C.T., Lalonde, S.V., Knudsen, A., Wang, X., Ossa, F.O., Pecoits, E., Smith, A.J., and Beukes, N.J. (2014a) Evidence for oxygenic photosynthesis half a billion years before the Great Oxidation Event. *Nature Geosci* 7:283-286.

Planavsky, N.J., Reinhard, C.T., Wang, X., Thomson, D., McGoldrick, P., Rainbird, R.H., Johnson, T., Fischer, W.W., and Lyons, T.W. (2014b) Low Mid-Proterozoic atmospheric oxygen levels and the delayed rise of animals. *Science* 346:635-638.

Planavsky, N.J., Cole, D.B., Isson, T.T., Reinhard, C.T., Crockford, P.W., Sheldon, N.D., and Lyons, T.W. (2018) A case for low atmospheric oxygen levels during Earth's middle history. *Emerging Topics Life Sci* 2:149-159.

Porter, S.M. (2016) Tiny vampires in ancient seas: evidence for predation via perforation in fossils from the 780–740 million-year-old Chuar Group, Grand Canyon, USA. *Proc Royal Soc B: Biolo Sci* 283:20160221.

Porter, S.M., Meisterfeld, R., and Knoll, A.H. (2003) Vase-shaped microfossils from the Neoproterozoic Chuar Group, Grand Canyon: a classification guided by modern testate amoebae. *J Paleontol* 77:409-429.

Qu, Y., Zhu, S., Whitehouse, M., Engdahl, A., and McLoughlin, N. (2018) Carbonaceous biosignatures of the earliest putative macroscopic multicellular eukaryotes from 1630 Ma Tuanshanzi Formation, north China. *Precambrian Res* 304:99-109.

Riedman, L.A. and Porter, S. (2016) Organic-walled microfossils of the mid-Neoproterozoic Alinya Formation, Officer Basin, Australia. *J Paleontol* 90:854-887.

Riedman, L.A., Porter, S.M., Halverson, G.P., Hurtgen, M.T., and Junium, C.K. Organic-walled microfossil assemblages from glacial and interglacial Neoproterozoic units of Australia and Svalbard. *Geology* 42:1011-1014.

Sahoo, S.K., Planavsky, N.J., Jiang, G., Kendall, B., Owens, J.D., Wang, X., Shi, X., Anbar, A.D., and Lyons, T.W. (2016) Oceanic oxygenation events in the anoxic Ediacaran ocean. *Geobiology* 14:457-468.

Samuelsson, J., Dawes, P.R., and Vidal, G. (1999) Organic-walled microfossils from the Proterozoic Thule Supergroup, northwest Greenland. *Precambrian Res* 96:1-23.

Satkoski, A.M., Beukes, N.J., Li, W., Beard, B.L., and Johnson, C.M. (2015) A redox-stratified ocean 3.2 billion years ago. *Earth Planet Sci Lett* 430:43-53.

Schachat, S.R., Labandeira, C.C., Saltzman, M.R., Cramer, B.D., Payne, J.L., and Boyce, C.K. (2018) Phanerozoic pO_2 and the early evolution of terrestrial animals. *Proc R Soc B* 285:20172631.

Scott, C.T., Bekker, A., Reinhard, C.T., Schnetger, B., Krapež, B., Rumble III, D., and Lyons, T.W. (2011) Late Archean euxinic conditions before the rise of atmospheric oxygen. *Geology* 39:119-122.

Sergeev, V.N., Knoll, A.H., and Vorob'Eva, N.G. (2011) Ediacaran microfossils from the Ura Formation, Baikal-Patom Uplift, Siberia: taxonomy and biostratigraphic significance. *J Paleontol* 85:987-1011.

Sergeev, V.N., Knoll, A.H., Vorob'eva, N.G., and Sergeeva, N.D. (2016) Microfossils from the lower Mesoproterozoic Kaltasy Formation, East European Platform. *Precambrian Res* 278:87-107.

Sergeev, V.N. and Schopf, J.W. (2010) Taxonomy, paleoecology and biostratigraphy of the late Neoproterozoic Chichkan microbiota of South Kazakhstan: The marine biosphere on the eve of metazoan radiation. *J Paleontol* 84:363-401.

Sharma, M. and Shukla, Y. (2012) Occurrence of helically coiled microfossil Obruchevella in the Owk Shale of the Kurnool Group and its significance. *J Earth Syst Sci* 121:755-768.

Shi, M., Feng, Q., Khan, M.Z., and Zhu, S. (2017) An eukaryote-bearing microbiota from the early Mesoproterozoic Gaoyuzhuang Formation, Tianjin, China and its significance. *Precambrian Res* 303:709-726.

Shi, W., Li, C., Luo, G., Huang, J., Algeo, T.J., Jin, C., Zhang, Z., and Cheng, M. (2018) Sulfur isotope evidence for transient marine-shelf oxidation during the Ediacaran Shuram Excursion. *Geology* 46:267-270.

Sperling, E.A., Wolock, C.J., Morgan, A.S., Gill, B.C., Kunzmann, M., Halverson, G.P., Macdonald, F.A., Knoll, A.H., and Johnston, D.T. (2015) Statistical analysis of iron geochemical data suggests limited late Proterozoic oxygenation. *Nature* 523:451-454.

Stanevich, A.M., Maksimova, E.N., Kornilova, T.A., Gladkochub, D.P., Mazukabzov, A.M., and Donskaya, T.V. (2009) Microfossils from the Arymas and Debengda formations, the Riphean of the Olenek Uplift: Age and presumable nature. *Strat Geol Corr* 17:20-35.

Strother, P.K. (2011) Surprisingly high biotic diversity in a Neoproterozoic shale. *GSA Annual Meeting in Minneapolis*.

Strother, P.K. (2012) Paleobiology of earliest Neoproterozoic lakes. *GSA Annual Meeting in Charlotte*.

Tang, Q., Pang, K., Xiao, S., Yuan, X., Ou, Z., and Wan, B. (2013) Organic-walled microfossils from the early Neoproterozoic Liulaobei Formation in the Huainan region of North China and their biostratigraphic significance. *Precambrian Res* 236:157-181.

Vidal, G. (1976) *Late Precambrian microfossils from the Visingso Beds in southern Sweden*. Universitetsforlaget.

Vidal, G. and Ford, T. D. (1985) Microbiotas from the late Proterozoic Chuar Group (northern Arizona) and Uinta Mountain Group (Utah) and their chronostratigraphic implications. *Precambrian Res* 28:349-389.

Vidal, G. and Siedlecka, A. (1983) Planktonic, acid-resistant microfossils from the upper Proterozoic strata of the Barents Sea region of Varanger Peninsula, East Finnmark, Northern Norway. *Norges Geologiske Undersokelse Bulletin* 71:45-79.

Vorob'eva, N.G., Sergeev, V.N., and Knoll, A.H. (2009) Neoproterozoic microfossils from the northeastern margin of the East European Platform. *J Paleontol* 83:161-196.

Wallace, M.W., Shuster, A., Greig, A., Planavsky, N.J., and Reed, C.P. (2017) Oxygenation history of the Neoproterozoic to early Phanerozoic and the rise of land plants. *Earth Planet Sci Lett* 466:12-19.

Xiao, S., Knoll, A.H., Yuan, X., and Pueschel, C.M. (2004) Phosphatized multicellular algae in the Neoproterozoic Doushantuo Formation, China, and the early evolution of florideophyte red algae. *Am J Botany* 91:214-227.

Xiao, S., Yuan, X., Steiner, M., and Knoll, A.H. (2002) Macroscopic carbonaceous compressions in a terminal Proterozoic shale: a systematic reassessment of the Miaohe biota, South China. *J Paleontol* 76:347-376.

Xiao, S., Shen, B., Tang, Q., Kaufman, A.J., Yuan, X., Li, J., and Qian, M. (2014a) Biostratigraphic and chemostratigraphic constraints on the age of early Neoproterozoic carbonate successions in North China. *Precambrian Res* 246:208-225.

Xiao, S., Zhou, C., Liu, P., Wang, D., and Yuan, X. (2014b) Phosphatized acanthomorphic acritarchs and related microfossils from the Ediacaran Doushantuo Formation at Weng'an (South China) and their implications for biostratigraphic correlation. *J Paleontol* 88:1-67.

Xunlai, Y., Jun, L., and Ruiji, C.A. (1999) Diverse metaphyte assemblage from the Neoproterozoic black shales of South China. *Lethaia* 32:143-155.

Yang, S., Kendall, B., Lu, X., Zhang, F., and Zheng, W. (2017) Uranium isotope compositions of mid-Proterozoic black shales: Evidence for an episode of increased ocean oxygenation at 1.36 Ga and evaluation of the effect of post-depositional hydrothermal fluid flow. *Precambrian Res* 298:187-201.

Yin, X., Yuan, X., Meng, F., and Hu, J. (2005) Protists of the Upper Mesoproterozoic Ruyang Group in Shanxi Province, China. *Precambrian Res* 141:49-66.

Yuan, X., Xiao, S., Li, J., Yin, L., and Cao, R. (2001) Pyritized chuarids with excystment structures from the late Neoproterozoic Lantian Formation in Anhui, South China. *Precambrian Res* 107:253-263.

Zhang, S., Wang, X., Wang, H., Bjerrum, C.J., Hammarlund, E.U., Costa, M.M., Connelly, J.N., Zhang, B., Su, J., and Canfield, D.E. (2016) Sufficient oxygen for animal respiration 1,400 million years ago. *Proc Natl Acad Sci USA* 113:1731-1736.

Zang, W. L. (1995) Early Neoproterozoic sequence stratigraphy and acritarch biostratigraphy, eastern Officer Basin, South Australia. *Precambrian Res* 74:119-175.

Zang, W. and Walter, M.R. (1992) *Late Proterozoic and Cambrian microfossils and biostratigraphy, Amadeus basin, central Australia*. (Association of the Australasian Palaeontologists).

Zhang, Z. (1997) A new Palaeoproterozoic clastic-facies microbiota from the Changzhougou Formation, Changcheng Group, Jixian, north China. *Geol Mag* 134:145-150.

Zhu, S., Zhu, M., Knoll, A.H., Yin, Z., Zhao, F., Sun, S., Qu, Y., Shi, M., and Liu, H. (2016) Decimetre-scale multicellular eukaryotes from the 1.56-billion-year-old Gaoyuzhuang Formation in North China. *Nature Comm* 7:1-8.
